# Associations between caregiver‐adolescent emodiversity and internalizing symptoms

**DOI:** 10.1111/jora.13041

**Published:** 2024-12-01

**Authors:** Jessica P. Lougheed, Colin F. Loveridge, Gizem Keskin, Nancy L. Sin

**Affiliations:** ^1^ Department of Psychology University of British Columbia Okanagan Kelowna British Columbia Canada; ^2^ Department of Psychology University of British Columbia Vancouver Vancouver British Columbia Canada

**Keywords:** internalizing symptoms, interpersonal emotions, parent‐adolescent relationships

## Abstract

Relationships between adolescents and primary caregivers play an important role in the development of internalizing (depressive and anxious) symptoms. We examined associations among caregiver‐adolescent emotions and their emodiversity (their breadth and frequency) with adolescents' and caregivers' internalizing symptoms. A total of 181 adolescents (aged 13–17 years old) and their primary caregivers participated in a 14‐day daily diary study between 2022 and 2023 where they reported their emotions at the end of each day. We used actor‐partner interdependence models to examine whether positive and negative emotions and their emodiversity predicted each individual's and their family member's internalizing symptoms. Primary results concentrated on the actor effects of adolescents' and caregivers' mean levels of positive and negative emotions and their own internalizing symptoms. Greater negative emotion and lower positive emotion were associated with greater internalizing symptoms, for both caregivers and adolescents. Caregivers who showed greater positive emodiversity also showed greater internalizing symptoms. In contrast, relatively few partner effects were observed. Our findings point to the need for more research on the role of interpersonal emotions in daily life as they relate to internalizing symptoms. Although our sample captured diversity in ethnicity, results may not generalize across levels of socioeconomic status, gender identity, and caregiver education.

## INTRODUCTION

Internalizing problems can affect the emotional, social, mental, and physical health of any person (Sands et al., 2022). Internalizing disorders are a grouping of overlapping symptom profiles such as anxiety, loneliness, depression, and sadness that internalize emotion and distress (Achenbach et al., 2016; Zahn‐Waxler et al., 2000). Individual differences in emotion‐related processes, such as emotional variability and emotion regulation skills, are associated with internalizing problems across ages, and adolescence is an especially important developmental period to examine these processes given age‐typical changes to emotionality (Hollenstein & Lougheed, 2013; Kuppens et al., 2012). Adolescents who develop internalizing symptoms are at an increased risk of recurrence, persistence, and/or increase in internalizing symptoms in adulthood (Janssen et al., 2021; Wetter & El‐Sheikh, 2012). Primary caregivers play a significant role in the socialization of adolescents' emotion and emotion regulation skills, and a large body of research demonstrates that caregiver‐adolescent emotion dynamics are associated with the internalizing problems of both adolescents and their primary caregivers (e.g., Lougheed & Hollenstein, 2016; Van der Giessen et al., 2014). We examined emotions experienced by adolescents and their primary caregivers in daily life, and how two features of positive and negative emotions—their mean levels and their diversity/frequency—were related to both adolescents' and caregivers' depressive and anxious symptoms.

Decades of research have demonstrated links between positive and negative emotions and internalizing symptoms. In general, depressive symptoms in both adolescents and adults are associated with greater levels of negative emotions, with specific negative emotions including sadness and anger in adolescents (Silk et al., 2003). The associations between positive emotions and depressive symptoms are complex, with some studies showing that depression is associated with lower levels of positive emotions (Werner‐Seidler et al., 2013), and others showing that depression may be associated with greater positive emotional responses to positive events (e.g., Nelson et al., 2020). Anxious symptoms are also associated with elevated negative and decreased positive emotion (Brown, 2007; Kashdan, 2007) but are perhaps not associated with positive emotional responses to positive events (Van Loo et al., 2023). Taken together, mean levels of positive and negative emotions are associated with both anxious and depressive symptoms in adolescents and adults, and anxiety and depression show both similarities and differences in terms of these associations. More recently, researchers have focused on variability in positive and negative emotions as they relate to internalizing problems to better differentiate anxiety and depression in terms of their associated emotion processes.

To date, the most common constructs used to capture variability in emotions include emotional lability, which captures the degree of fluctuations in specific emotions over time (e.g., Silk et al., 2003) and inertia, which reflects the extent to which emotions are resistant to change over time (Kuppens et al., 2012). These constructs, which index variations (or lack thereof) in single emotion states over time, have yielded valuable insights into how emotions in daily life are associated with internalizing symptoms in both adolescents and adults (Houben et al., 2015). For example, greater lability of anger, sadness, and anxiety are associated with depressive symptoms in adolescents (Silk et al., 2003), and greater emotional inertia predicts the onset of depressive episodes (Kuppens et al., 2012). The complementary concept of emodiversity (Quoidbach et al., 2014) captures the variations in the specific *types* of emotions experienced. Emodiversity (Quoidbach et al., 2014) refers to the breadth (i.e., number of different emotions) and frequency of emotions that one experiences and may indicate the ability to adapt emotional responses to changing situational contexts.

**FIGURE 1:**
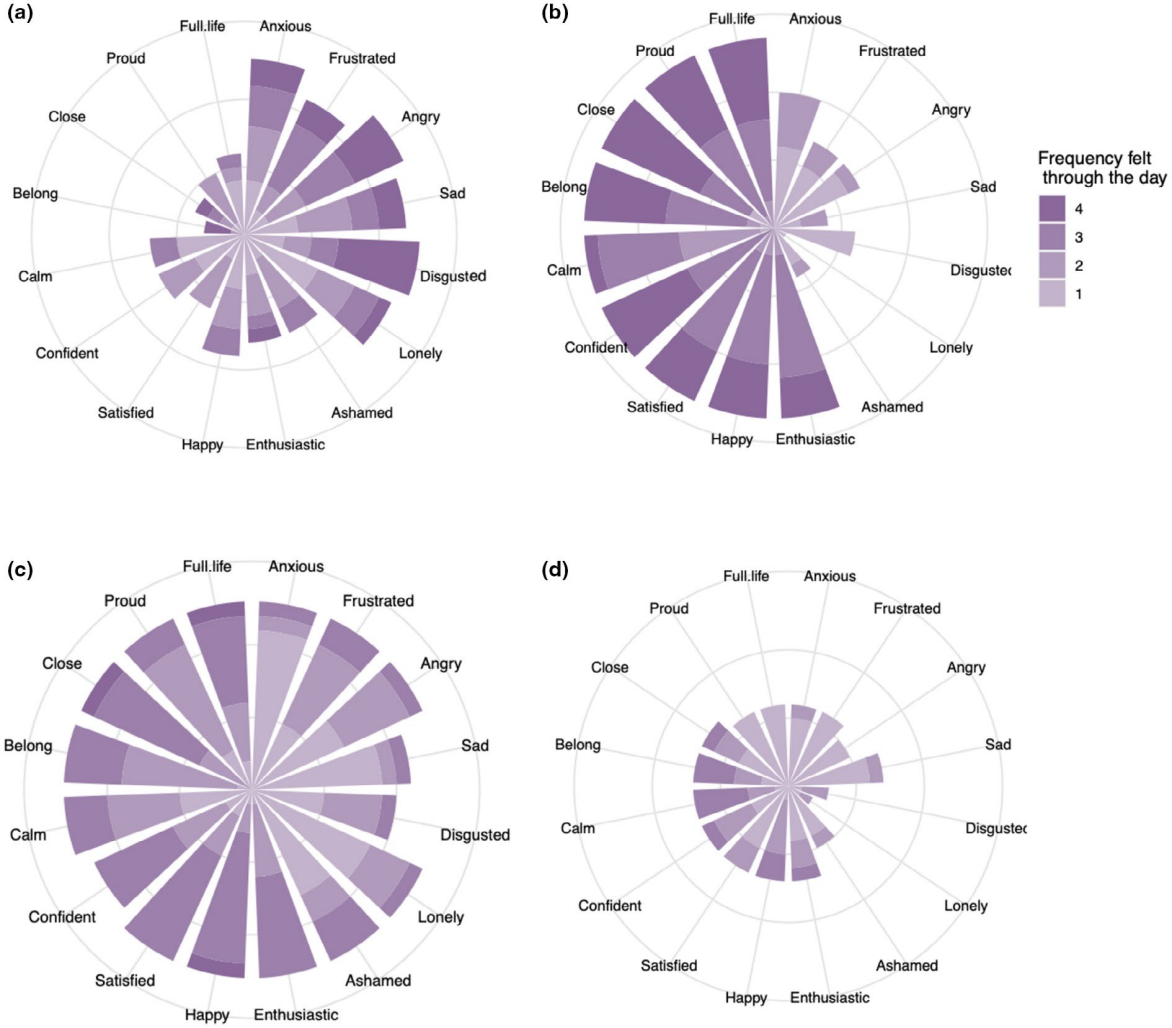
Visual representations of different combinations of high and low negative and positive emodiversity from four participants. These plots show positive and negative emotion data for four different participants from the current study. The plots show participants with: High negative emodiversity and low positive emodiversity (Panel a), low negative emodiversity and high positive emodiversity (Panel b), high negative and positive emodiversity (Panel c), and low negative and positive emodiversity (Panel d).

Figure 1 illustrates emodiversity in four different study participants with different combinations of high and low positive and negative emodiversity based on daily reports of 16 different emotions. The length of the “spokes” shows the number of times each emotion was reported across days, and the colors indicate the frequency reported within days. Figure 1a shows an individual with high negative emodiversity (by endorsing a breadth of different negative emotions and relatively high frequencies within each day) and low positive emodiversity. Figure 1b shows an individual with high positive emodiversity (by endorsing a breadth of different positive emotions and relatively high frequencies within each day) and low negative emodiversity. Figure 1c shows an individual with high emodiversity across all emotions (by endorsing each emotion measured with relatively high frequencies within each day). Figure 1d shows an individual with low emodiversity across all emotions (low endorsement of different emotions and low frequences reported within days). The overarching goal of the current study was to examine how mean levels and emodiversity of positive and negative emotions are associated with depressive and anxious symptoms in the context of caregiver‐adolescent relationships.

## THE ASSOCIATIONS BETWEEN EMODIVERSITY AND WELL‐BEING

The first examination of emodiversity (Quoidbach et al., 2014) involved measuring it from emotions assessed at a single time point and demonstrated that greater positive and negative emodiversity were uniquely associated with better mental and physical health, above the contribution of mean levels of positive and negative emotions. Benson et al. (2017) extended the measurement of emodiversity to intensive longitudinal data obtained from daily reports of individuals' emotions. Research examining emodiversity in daily life has added nuance to our understanding of when, and for whom, emodiversity is associated with well‐being, although this concept is not without critique (see Brown & Coyne, 2017 for critique and Quoidbach et al., 2018 for rebuttal). To summarize, critiques of this approach are related to whether the use of the statistical metric, Shannon's entropy, is appropriate to the typical measures of emotion used in psychology (Brown & Coyne, 2017). Quoidbach et al. (2018) demonstrated the appropriate application of this measure to many types of psychological constructs over the last couple of decades, and demonstrated conceptual ties between the originally‐developed use of this metric to capture ecological diversity and emotional diversity.

Positive and negative emodiversity are uniquely associated with mental health and wellbeing. In adolescents, negative emodiversity, but neither positive nor total emodiversity (i.e., calculated across both positively and negatively valenced emotions), predicted emotional eating behaviors, which are associated with difficulties regulating emotions (Heshmati et al., 2023). Moreover, mean levels of positive and negative emotions are an important context through which emodiversity is associated with mental and physical health outcomes. For example, in a community‐based sample of adults, greater negative emodiversity was associated with better physical health for individuals experiencing higher mean levels of negative emotion (Benson et al., 2017), and greater positive emodiversity was associated with lower levels of systemic inflammation (an indicator of better physical health; Ong et al., 2018). In a sample of undergraduate students, lower negative emodiversity was associated with greater depressive and anxious symptoms in the context of high mean levels of negative emotion, whereas greater negative emodiversity in the context of high mean levels of negative emotion was associated with greater well‐being (Forster & Lougheed, 2024). Taken together, experiencing a breadth of positive and negative emotions in daily life—especially among those experiencing high levels of negative emotions—may signal functional adaptation to changing contextual demands (Ong et al., 2018). One characteristic of internalizing problems is context insensitivity (Kashdan & Rottenberg, 2010), which includes difficulties adjusting emotions and their related processes to changes in daily contexts. More research is needed to understand associations between emodiversity and internalizing problems in different populations.

## EMODIVERSITY IN CAREGIVER‐ADOLESCENT DYADS

To date, research on emodiversity in daily life and its associations with well‐being has been from an intrapersonal perspective. However, interpersonal perspectives on emotion emphasize that emotions and their dynamics are enacted in the context of social relationships (Butler, 2011; Lougheed, 2020). In close dyadic relationships, emotions emerge through the dynamics of the temporal interpersonal emotion system (TIES); (Butler, 2011), whereby the interactions within and between each person's intrapersonal emotion dynamics interweave to form an *interpersonal* system (Butler, 2011; Lougheed, 2020). Through repetition, emotion dynamics on short time scales (e.g., moment to moment, day to day) may coalesce into longer‐standing traits and characteristics such as internalizing symptoms that then may constrain how emotion dynamics unfold on short time scales (Lougheed, 2020). Caregivers and adolescents are nested within their own, and each other's, micro‐scale emotion dynamics and macro‐scale psychosocial adjustment such as internalizing symptoms. Thus, there may be mutual influences between one person's emotion dynamics and their partner's internalizing symptoms. For example, an adolescent's depressive symptoms may be associated with their caregiver's difficulty experiencing and expressing a range of emotions in daily life, and vice versa.

To date, no research has examined emodiversity in an interpersonal context. For caregiver‐adolescent dyads specifically, some research has examined how other aspects of emotional variability are related to internalizing symptoms. Several features of adolescence emphasize the importance of examining emotional variability in caregiver‐adolescent dyads. First, although no new emotions emerge in adolescence, the intensity and variability of emotion is heightened (Granic et al., 2003; Hollenstein & Lougheed, 2013), centering emotion regulation as a key developmental task at this stage (Rosenblum & Lewis, 2006). Second, adolescents are at greater risk for developing internalizing symptoms than they were in childhood, and their evolving emotion dynamics with their primary caregivers play a role in that too (Lougheed, 2020). For example, caregiver‐adolescent dyads who showed lower emotional variability during conflicts were more likely to develop internalizing symptoms, for both mothers and their adolescents, 5 years later (Van der Giessen et al., 2014). Third, emotion socialization processes, such as how caregivers model the expression and regulation of specific types of emotions, help shape adolescents' own emotional experiences (Morris et al., 2018). It is therefore possible that emodiversity—experiencing a broad range of emotions—is to some extent shared between caregivers and adolescents and related to their internalizing symptoms. Our goal was to examine how caregivers' and adolescents' positive and negative emodiversity in daily life are associated with their own and each other's internalizing symptoms.

## THE CURRENT STUDY

We explored the associations between caregivers' and adolescents' emodiversity and internalizing symptoms with research questions regarding the actor effects (individual emodiversity predicting their own internalizing symptoms) and partner effects (individual emodiversity predicting their family member's internalizing symptoms). Previous research has shown that mean levels of positive and negative emotions are important to consider when examining associations between positive and negative emodiversity and wellbeing (Benson et al., 2017; Forster & Lougheed, 2024). We examined each individual's positive and negative emodiversity, and the interactions of positive and negative emodiversity with mean levels of positive and negative emotions, respectively, in predicting their own and each other's anxious and depressive symptoms. We examined these associations for both anxious and depressive symptoms given that current the links between emotions and these internalizing problems share some similarities but also have some differences (Bylsma, 2021). For example, although both depression and anxiety are associated with elevated negative emotions, they may differ in terms of the specific types of negative emotions experienced and how they are regulated (Bylsma, 2021). In addition, depressive symptoms may be further distinguished from anxious symptoms as they include the unique symptom of anhedonia, which refers to a pervasive difficulty feeling pleasurable emotional states (De Fruyt et al., 2020), even though anxiety may also involve difficulties experiencing positive emotions (Kashdan & Steger, 2006). Thus, there is more to learn about how anxious and depressive symptoms may be distinguished by emotions and so we examined these two categories of symptoms separately. Our research questions were whether: (1) caregiver and adolescent mean levels of positive and negative emotions are associated with their own (actor effect) and each other's (partner effect) depressive and anxious symptoms; (2) caregiver and adolescent positive and negative emodiversity are associated with their own (actor effect) and each other's (partner effect) depressive and anxious symptoms; and (3) caregiver and adolescent positive and negative emodiversity interact with mean levels of positive and negative emotion (respectively) in predicting depressive and anxious symptoms.

Our preregistered hypotheses and analysis plan are available here. We hypothesized that individual caregiver and adolescent mean negative emotion would be positively associated with their own depressive and anxious symptoms (actor effects) and each other's depressive and anxious symptoms (partner effects; Benson et al., 2017; Maciejewski et al., 2014; Quoidbach et al., 2014). Next, we hypothesized that caregivers' and adolescents' mean positive emotion would be negatively associated with their own depressive and anxious symptoms (actor effects) and each other's depressive and anxious symptoms (partner effects; Benson et al., 2017; Quoidbach et al., 2014). Due to the lack of research to date on emodiversity in caregiver‐adolescent dyads and its relationship with respect to caregiver and adolescent internalizing symptoms, and in line with current theoretical perspectives that the associations between emotional variability and mental health outcomes likely vary according to what is considered optimal emotion levels in specific contexts (Jenkins et al., 2024), we did not have specific hypotheses regarding the directionality of the emodiversity associations. As a result, we hypothesized that individual caregiver and adolescent emodiversity (positive or negative) would be associated with their own depressive and anxious symptoms (actor effects) and each other's depressive and anxious symptoms (partner effects); however, we did not hypothesize the specific direction of the effects. In addition, we hypothesized that individuals' mean positive and negative emotion and their respective emodiversity would interact in predicting depressive and anxious symptoms in line with previous findings (Forster & Lougheed, 2024).

## METHOD

We collected 14 days of daily diary data from a community sample of adolescents and their primary caregivers. Prior to the daily diaries, we collected information from a one‐time survey on participant demographics and internalizing symptoms. Then, participants reported each day on the emotions they experienced.

### Participants

The participants were a sample of 181 adolescents and their primary caregivers. Analyses were conducted on 175 of these dyads as the excluded dyads met one or more of the exclusion criteria developed to ensure reliability of data, discussed further below. Our sample size was determined by the maximum number of dyads we were able to enroll in this study during the data collection period. Participants were recruited from within British Columbia, Canada between January 16, 2022, and August 16, 2023. Participant recruitment occurred through location‐restricted and age demographic‐targeted advertisements on Instagram, physical flyers posted in community locations in Kelowna, BC (e.g., coffee shops, recreation centers), and three private schools distributed flyers for adolescents to take home to their families.

Adolescents' ages ranged from 13 to 17 years old (*M* = 14.37, *SD* = 1.27). Primary caregivers' ages ranged from 30 to 57 years old (*M* = 45.83, *SD* = 4.71). Among adolescent participants, almost half of the sample (44%) identified their gender as “Boy/Man” or “Girl/Woman” (49%). Almost all caregivers (93%) reported their gender as “Woman”. See Table S1 for full details on participant gender identity.

Many adolescent participants identified their ethnicity as “White” (39%) or endorsed multiple ethnicity options (33%). Approximately half (53%) of caregivers reported their ethnicity as “White” (53%), and several caregivers endorsed multiple ethnicity options (15%). See Table S1 for full details on participant ethnicity.

The sample of caregivers showed relatively high levels of education, with almost half (45%) of caregivers reporting their highest level of education as “Bachelor's Degree” and 34% as “Graduate Degree”. The sample of caregivers also showed relatively high household income, with many reporting their household income as above $150,000 per year (33%) or between $100,000 and $150,000 per year (31%). Most caregivers reported their relationship status as married (70%). Almost all (98%) caregivers reported being the biological parent of their participating adolescent. The remaining relationship types caregivers reported (all <1%) were as the parent's partner (living together), foster parent, or as a relationship type not listed. See Table S1 for full details on caregiver education, household income, and relationship status.

### Procedure

Approval for these procedures was obtained from the Behavioral Research Ethics Board at the University of British Columbia Okanagan prior to data collection (Protocol H21‐01599). The study procedures consisted of two parts. After caregivers provided informed consent for their own and their adolescents' participation, adolescents then provided assent to participate. Then, in Part 1, both caregivers and adolescents completed a one‐time questionnaire via a free mobile survey app called ExpiWell (Tay, 2020) asking about demographic information, developmental status, emotional tendencies, mental health, and relationship characteristics. Upon the completion of Part 1, both caregivers and adolescents began Part 2 of the study on the same day after an instructional call with the project research assistant. In Part 2, at the end of the day via ExpiWell, caregivers and adolescents provided an independent self‐report of their positive and negative emotions and various aspects of their interactions with their participating family member (e.g., positive experiences, conflicts) that day. Participants received a reminder notification after 90 minutes. We initially started with a 21‐day design for Part 2 but ended up reducing it to a 14‐day design to minimize participant burden and maximize our recruitment efforts.

Participants received compensation in the form of an e‐gift card to a location of their choosing from several options upon completion of Part 2. Different honorarium amounts were provided in accordance with adjustments to our procedures to maximize participant recruitment. Participants (*n* dyads = 5) who were recruited between January 16, 2022 and July 4, 2022 received an honorarium in the amount of $5 for Part 1 and $1 per completed prompt (up to 21 days) for Part 2. Participants (*n* dyads = 4) who were recruited between July 4, 2022 and October 17, 2022 received $5 for Part 1 and $1.50 per prompt (up to 14 days) for Part 2. Participants (*n* dyads = 166) who were recruited between October 17, 2022 and August 16, 2023 received $7 for Part 1 and $2 per completed prompt (up to 14 days) for Part 2.

Participants who met the following criteria were excluded prior to analyses. First, participants whose participating family member completed Part 1 of the study but did not participate in Part 2 were excluded. Second, participants who do not have at least 6 days of data for reliable calculation of emodiversity were excluded (Benson et al., 2017). Third, participants whose participating family member did not have sufficient data from Part 2 were excluded. After these exclusions, the final sample used in analyses consisted of 175 dyads. Compliance was high, with adolescents completing 84% of prompts and caregivers completing 88% of prompts. The mean number of daily responses was 12.01 for adolescents and 12.55 for caregivers.

### Measures

#### Self‐reported anxious symptoms

Both caregivers' and adolescents' self‐reported anxious symptoms were measured with the Beck Anxiety Inventory (Beck et al., 1988) during Part 1 of the study. On the questionnaire, participants rated their responses to 21 items (e.g., “Numbness and tingling” and “Fear of losing control”) indicating the degree to which they are bothered by each symptom on a scale from 0 (*Not at all*) to 3 (*Severely, I could barely stand it*). Internal consistency was satisfactory, with *α* = .91 for caregivers and α = .94 for adolescents. The mean of each participant's reports on all anxious items was calculated. Higher values indicate greater anxious symptoms.

#### Self‐reported depressive symptoms

During Part 1 of the study, caregivers self‐reported depressive symptoms with the Beck Depression Inventory, second edition (BDI‐II; Beck et al., 1996) which contains 21 groups of statements regarding the caregiver's feelings. To meet the requirements of the research ethics board, we omitted the item pertaining to suicidality. Participants rated their responses to the 20 groups of statements by selecting which best described the way they have been feeling during the past 2 weeks on a scale from 0 (e.g., *I feel the same about myself as ever*) to 3 (*I dislike myself*). Internal consistency was high (α = .89). The mean of each caregiver's responses to all items was calculated, with higher values indicating greater depressive symptoms.

During Part 1 of the study, adolescents' self‐reported depressive symptoms were measured with the Child Depression Inventory (Kovacs, 1985) which contains 26 groups of statements regarding the adolescents' feelings. Participants rated their responses for each item by indicating which response best indicated their experience from the past 2 weeks, for example, from 0 (*I do not feel alone*) to 3 (*I feel alone all the time*). The mean of each participant's responses to all items was calculated as the adolescents' self‐reported depressive symptoms, with higher values indicating greater depressive symptoms. Internal consistency was high (*α* = .86).

#### Daily negative and positive emotion

On each day of Part 2, participants responded to a questionnaire that contained 16 emotion‐word items for participants to rate their daily emotion experiences. This measure was adapted from the National Study of Daily Experiences (Charles et al., 2019). Participants rated their responses to the question, “Below is a list of words describing different feelings. How well does each word describe how you felt today?” on a scale from 0 to 4 (0 = *None of the time*, 1 = *A little of the time*, 2 = S*ome of the time*, 3 = *Most of the time*, 4 = *All of the time*): anxious, sad, angry, frustrated, enthusiastic, happy, disgusted, satisfied, confident, calm, like you belong, close to others, lonely, ashamed, proud, and full of life. Items indicating negative emotion were anxious, sad, angry, frustrated, disgusted, lonely, and ashamed.

##### Mean negative emotion

Each participant's responses to all negative emotion items (anxious, sad, angry, frustrated, disgusted, lonely, and ashamed) were calculated across days. Within each day, the seven negative emotion items were averaged to produce a composite mean negative emotion score. Subsequently, these daily mean scores were averaged across the measurement occasions to obtain each participant's mean negative emotion score. Participants' mean negative emotion represents the average magnitude of the negative emotions the participant experienced across all daily reports, with higher values indicating a greater negative emotion. We calculated the between‐person reliability for these items using best practices for intensive longitudinal data (Bolger & Laurenceau, 2013). The between‐person reliability for the negative emotion items was good for adolescents (0.71) and caregivers (0.73).

##### Negative emodiversity

We calculated the Gini coefficient as seen in Equation 1, as a measure of negative emodiversity (Benson et al., 2017). In this equation, *c*_*ij*_ represents each emotion experience within a range of *j* = 1 to *m* number of emotion categories reported from each individual (*i*), indexed in non‐decreasing order (*c*_*ij*_ *< = c*_*ij + 1*_); Benson et al. (2017). Scores on the Gini coefficient range from 0 (reported experiencing one single emotion) to 1 (reported experiencing all possible emotions), with higher values indicating more diverse negative emotions. The Gini coefficient indicates both the number of different emotions reported (richness) as well as the relative abundance (frequency felt throughout each day) of different emotions across reports in terms of their proportion (Quoidbach et al., 2014).

(1) $$\text{Gini}=1-\left(\left(\frac{2\sum_{j=1}^{m}{\mathrm{jc}}_{\mathrm{ij}}}{m\sum_{j=1}^{m}c_{\mathrm{ij}}}\right)-\frac{m+1}{m}\right).$$

##### Mean positive emotion

Each participant's reports on all positive emotion items (enthusiastic, happy, satisfied, confident, calm, like you belong, close to others, proud, and full of life) were calculated across days. Within each day, the nine positive emotion items were averaged to produce composite daily mean positive emotion. Subsequently, these daily mean scores were then averaged across the measurement occasions to obtain each participant's mean positive emotion. Participants' mean positive emotion represents the average magnitude of positive emotion the participant experienced across all daily reports, with higher values indicating greater levels of positive emotion. The between‐person reliabilities for adolescent (0.80) and caregiver (0.83) positive emotion items were good.

##### Positive Emodiversity

We calculated the Gini coefficient (Equation 1) to obtain the positive emodiversity variable (Benson et al., 2017). Participants' positive emodiversity scores range from 0 to 1, with higher values indicating more diverse positive emotions.

### Data analysis plan

#### Statistical models

**FIGURE 2:**
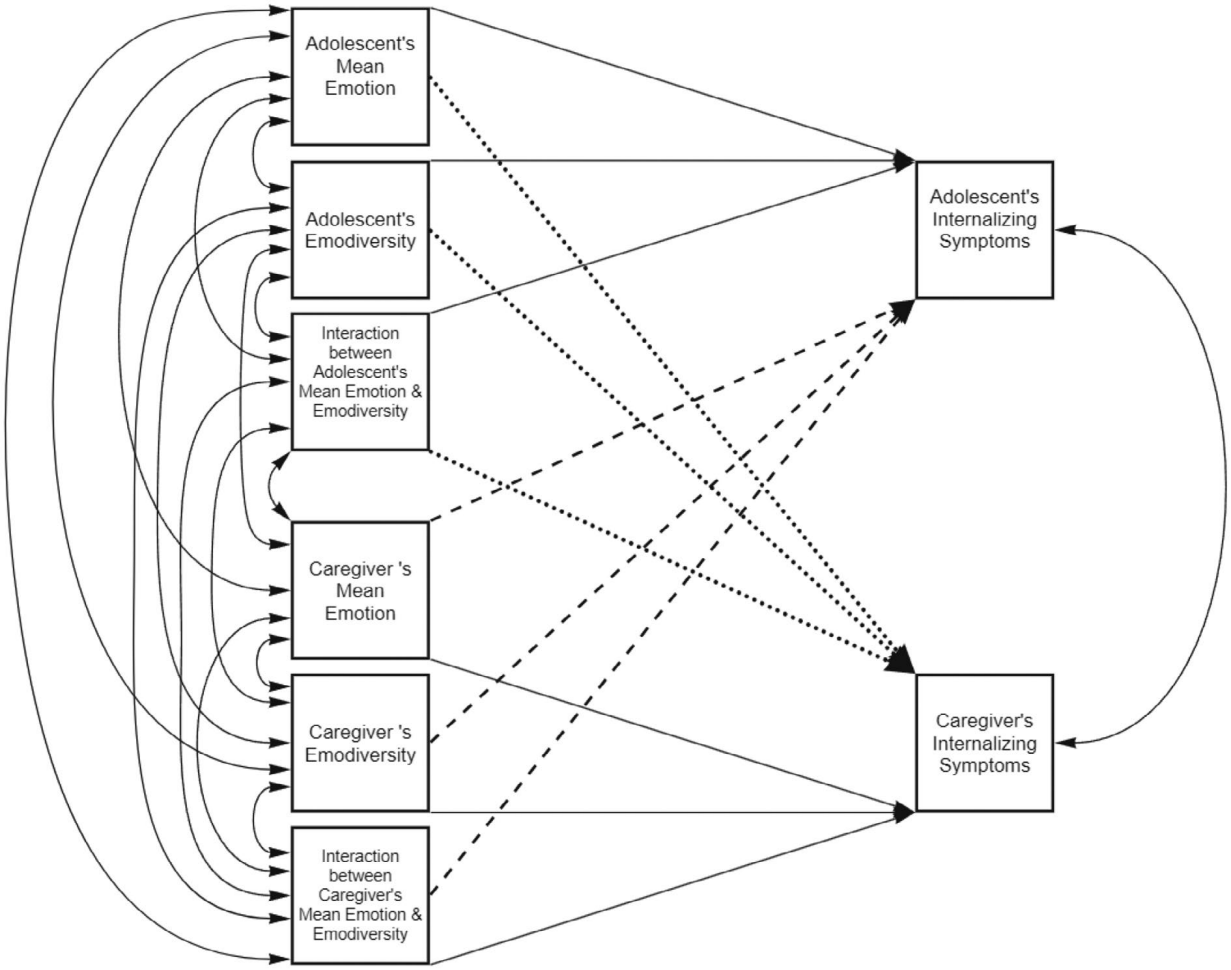
Conceptual model for the actor‐partner interdependence models examining associations among emotion variables and internalizing symptoms. single‐headed arrows indicate causal or predictive paths. double‐headed arrows indicate correlations.

Hypotheses were tested with four parallel bivariate actor–partner interdependence models (APIM) using the structural equation modeling (SEM) approach (Cook & Kenny, 2005). In each model, caregivers' and adolescents' mean emotion and emodiversity (negative or positive) were used as predictors of their respective outcome variables: Caregiver and adolescent anxious or depressive symptoms. Each of the four models examined one combination of negative or positive emotion variables (mean, emodiversity, and their interaction) as predictors of caregiver and adolescent anxious or depressive symptoms. To explore possible gender differences, we used multigroup analysis to explore paths separately by adolescent gender for those who identified as boys or girls. Due to the small number of adolescents (*n* = 4) who reported gender identities as nonbinary, gender non‐conforming, or questioning, we were not able to include these identities in analyses. The APIM using the SEM approach allowed us to explicitly model the dependence between family members' internalizing symptoms (Cook & Kenny, 2005). Figure 2 shows a conceptual model of associations between mean emotion, emodiversity, the interaction between emodiversity and mean emotion, and internalizing problems. Based on our research questions, we estimated each path of the conceptual model, which resulted in a just‐identified model estimation. We needed to estimate a series of just‐identified models to answer our research questions after we accounted for both the correlations among the exogenous variables and the correlations among the endogenous variables. Setting specific correlations between exogenous variables to zero resulted in poor model fit as expected due to associations among the exogenous variables, thus, our final models are just identified. A just‐identified model includes an equal number of known and free parameters in the model so that it has a zero degree of freedom. Hence, the model fit indices of just‐identified models show a perfect fit due to having zero degrees of freedom, which is a feature of this type of model rather than an indication of model fit (Raykov et al., 2013). Hence, we did not report the model fit indices. Maximum likelihood with robust standard errors (MLR) was used to account for the missingness and non‐normality of the data in Mplus version 8.8 (Muthén & Muthén, 2017).

### Transparency and openness

We report how we determined our sample size, all data exclusions, all manipulations, and all measures in the study, and the study follows JARS (Appelbaum et al., 2018). Data and analysis code are available here. This study's design, hypotheses, and analysis plan were preregistered while data were being collected but prior to accessing the data. Our final procedures differed from our preregistered plan in a few ways. First, our preregistration stated that we would finish collecting data in March 2023, but we were able to continue collecting data through August 2023 to maximize our sample size. Second, we added exploratory analyses regarding differences between adolescents and caregivers on key variables which were not preregistered. Third, we updated our language from the preregistration from “mood variability” to “emodiversity” to map onto other studies using the same methods (e.g., Benson et al., 2017; Quoidbach et al., 2014). Fourth, we used multigroup analyses to explore differences between adolescent boys and girls.

## RESULTS

Descriptive statistics of all variables were examined prior to analysis. Means, standard deviations, ranges, and bivariate correlations between all variable pairs are shown in Table 1. In general, variables were significantly correlated in the expected directions. For example, all bivariate correlations among caregiver variables were significantly correlated (e.g., negative emotion variables negatively correlated with positive emotion variables; internalizing symptoms negatively correlated with positive emotion variables). Correlations among adolescent variables and between adolescent and caregiver variables were less consistently significant (see Table 1). Table S2 shows correlations among study variables and demographics, and Table S3 shows descriptive statistics for study variables by adolescent gender identity.

**TABLE 1:** Descriptive statistics table for emotion and internalizing symptoms variables.

Variable	1.	2.	3.	4.	5.	6.	7.	8.	9.	10.	11.	12.	Means (*SD*)
1. Caregiver mean positive emotion	—												2.15 (0.54)
2. Caregiver mean negative emotion	−.54**	—											0.67 (0.35)
3. Caregiver positive emotion diversity	.72**	−.25**	—										0.89 (0.07)
4. Caregiver negative emotion diversity	−.36**	.72**	−.09	—									0.59 (0.14)
5. Caregiver depressive symptoms	−.47**	.64**	−.27**	.49**	—								0.49 (0.44)
6. Caregiver anxious symptoms	−.32**	.58**	−.10	.43**	.61**	—							0.40 (0.39)
7. Adolescent mean positive emotion	.20*	−.09	.17*	−.01	−.18*	−.02	—						2.04 (0.62)
8. Adolescent mean negative emotion	−.11	.13	−.14	.05	.16	.10	−.52**	—					0.90 (0.39)
9. Adolescent positive emotion diversity	.21**	−.06	.21**	−.02	−.24**	.02	.69**	−.08	—				0.87 (0.09)
10. Adolescent negative emotion diversity	−.10	.12	−.08	.11	.13	.07	−.32**	.73**	.04	—			0.72 (0.13)
11. Adolescent depressive symptoms	.00	.03	−.05	.01	.19*	.03	−.63**	.61**	−.41**	.35**	—		1.47 (0.30)
12. Adolescent anxious symptoms	−.17**	.15	−.17*	.03	.22**	.28***	−.33**	.49**	−.16*	.37**	.56**	—	0.88 (0.61)

*Note*: * indicates *p* < .05; ** indicates *p* < .01.

The assumptions of the SEM approach to APIMs include univariate and multivariate normality, linearity among pairs of variables, and the absence of multicollinearity. We examined univariate normality among predictors and found several indications of non‐normality among the positive and negative emodiversity measures. Thus, because the assumption of normality was not met, we used the MLR estimator to account for the non‐normality of the data. We found no evidence of curvilinear relationships among the scatterplots between predictor variables.

Next, the bivariate correlations among predictors were examined against the criteria >.80 to indicate possible multicollinearity. Bivariate correlations among all variable pairs were ≤.80. Additionally, we conducted exploratory *t*‐tests (not preregistered) on the outcome variables—anxious and depressive symptoms—between cases with and without missing data. All of these *t*‐tests were not significant (*p*s > .05), indicating no significant difference between participants with missing and non‐missing data on outcome variables. The emotion variables (mean emotion and emodiversity) were calculated only for individuals who do not meet the previously mentioned exclusion criteria and thus contain no missing data.

We also ran exploratory (not preregistered) two‐tailed paired‐sample *t*‐tests to examine whether adolescents and caregivers differed from each other on mean emotion levels and emodiversity. We did not find a significant difference in mean positive emotions, *t*(174) = −1.94, *p* = .05. Adolescents reported significantly greater negative emotions on average than caregivers, *t*(174) = 6.26, *p* < .001. Caregivers reported on average significantly greater positive emodiversity than adolescents, *t*(174) = −2.47, *p* = .01. Finally, adolescents had on average greater negative emodiversity than caregivers, *t*(172) = 9.64, *p* < .001.

### Actor and partner effects of emotion on internalizing symptoms

Table 2 presents the results of the APIM paths that we had hypotheses about for all models. Table S5 (see Supplemental Material) presents the results for all the paths estimated. In addition, we ran multigroup analyses on the same models to explore potential gender differences between adolescents who identified as boys and girls (see Tables S6 through S8). While several differences were observed between our primary results reported below and these supplementary models comparing boys and girls, we caution against the interpretation of the results of the multigroup analysis given the relatively low sample size and the generally small effects that were observed. In addition, we had to exclude the 4 adolescents who identified with a nonbinary/genderqueer/questioning gender identity to be able to run these multigroup models.

**TABLE 2:** Standardized results for actor‐partner interdependence models predicting internalizing symptoms from emotion and emodiversity.

	Effect on Adolescent's internalizing symptoms					Effect on Caregiver's internalizing symptoms				
	*β*	*SE*	*p*	*β* 95% CI		*β*	*SE*	*p*	*β* 95% CI	
				Low	High				Low	High
Model 1: Negative emotion, emodiversity, and anxious symptoms										
Adolescent mean negative emotion	0.49	0.10	<.001	0.32	0.65	0.03	0.09	.700	−0.11	0.18
Adolescent negative emodiversity	−0.02	0.11	.831	−0.21	0.16	0.00	0.10	.965	−0.17	0.16
Adolescent negative emotion × emodiversity	−0.06	0.07	.414	−0.17	0.06	0.07	0.07	.345	−0.05	0.19
Caregiver mean negative emotion	0.24	0.13	.061	0.03	0.45	0.55	0.12	<.001	0.35	0.75
Caregiver negative emodiversity	−0.16	0.11	.143	−0.34	0.02	0.04	0.11	.711	−0.14	0.22
Caregiver negative emotion × emodiversity	−0.07	0.09	.470	−0.22	0.09	0.01	0.09	.926	−0.14	0.15

	*β*	*SE*	*p*	*β* 95% CI		*β*	*SE*	*p*	*β* 95% CI	
				Low	High				Low	High
Model 2: Positive emotion, emodiversity, and anxious symptoms										
Adolescent mean positive emotion	−0.41	0.11	<.001	−0.58	−0.22	−0.09	0.10	.355	−0.26	0.07
Adolescent positive emodiversity	0.15	0.16	.358	−0.11	0.40	0.26	0.14	.064	0.03	0.49
Adolescent positive emotion × emodiversity	−0.02	0.11	.858	−0.19	0.16	0.18	0.10	.055	0.03	0.34
Caregiver mean positive emotion	−0.07	0.11	.510	−0.25	0.11	−0.52	0.10	<.001	−0.68	−0.35
Caregiver positive emodiversity	0.00	0.12	.989	−0.20	0.20	0.32	0.11	.002	0.15	0.50
Caregiver positive emotion × emodiversity	0.13	0.10	.169	−0.03	0.29	0.09	0.09	.308	−0.05	0.23

	*β*	*SE*	*p*	β 95% CI		*β*	*SE*	*p*	*β* 95% CI	
				Low	High				Low	High
Model 3: Negative emotion, emodiversity, and depressive symptoms										
Adolescent mean negative emotion	0.71	0.12	<.001	0.51	0.92	0.03	0.11	.746	−0.14	0.21
Adolescent negative emodiversity	−0.14	0.17	.394	−0.41	0.13	−0.01	0.11	.951	−0.20	0.18
Adolescent negative emotion × emodiversity	−0.03	0.11	.770	−0.21	0.15	0.02	0.07	.814	−0.09	0.12
Caregiver mean negative emotion	−0.09	0.15	.564	−0.33	0.16	0.63	0.13	<.001	0.42	0.63
Caregiver negative emodiversity	0.07	0.11	.548	−0.12	0.25	0.05	0.10	.585	−0.11	0.22
Caregiver negative emotion × emodiversity	0.03	0.08	.720	−0.10	0.15	−0.05	0.06	.814	−0.15	0.06

	*β*	*SE*	*p*	*β* 95% CI		*β*	*SE*	*p*	*β* 95% CI	
				Low	High				Low	High
Model 4: Positive emotion, emodiversity, and depressive symptoms										
Adolescent mean positive emotion	−0.75	0.09	<.001	−0.89	−0.60	−0.05	0.11	.636	−0.23	0.13
Adolescent positive emodiversity	0.16	0.11	.138	−0.02	0.34	0.10	0.13	.471	−0.12	0.32
Adolescent positive emotion × emodiversity	0.12	0.12	.296	−0.07	0.31	0.26	0.15	.094	0.00	0.51
Caregiver mean positive emotion	0.11	0.09	.211	−0.04	0.26	−0.55	0.12	<.001	−0.74	−0.36
Caregiver positive emodiversity	0.06	0.09	.492	−0.09	0.21	0.25	0.11	.028	0.06	0.44
Caregiver positive emotion × emodiversity	0.12	0.09	.171	−0.02	0.26	0.14	0.10	.162	−0.03	0.31

Abbreviations: CI, confidence interval; SE, standard error.

#### Model 1: Negative emotion variables and anxious symptoms

In predicting adolescent anxious symptoms, greater adolescent mean negative emotion was associated with greater adolescent anxious symptoms. In predicting caregiver anxious symptoms, greater caregiver negative emotion was associated with greater caregiver anxious symptoms. All other variables predicting adolescent and caregiver anxious symptoms were not significant. Regarding the multigroup analyses, greater levels of girls' and caregivers' mean negative emotions were associated with greater levels of adolescent girls' anxious symptoms (see Table S6). In addition, caregivers who participated with boys showed greater anxious symptoms when their boys showed high levels of mean negative emotion and high negative emodiversity (see Figure S1).

#### Model 2: Positive emotion variables and anxious symptoms

**FIGURE 3:**
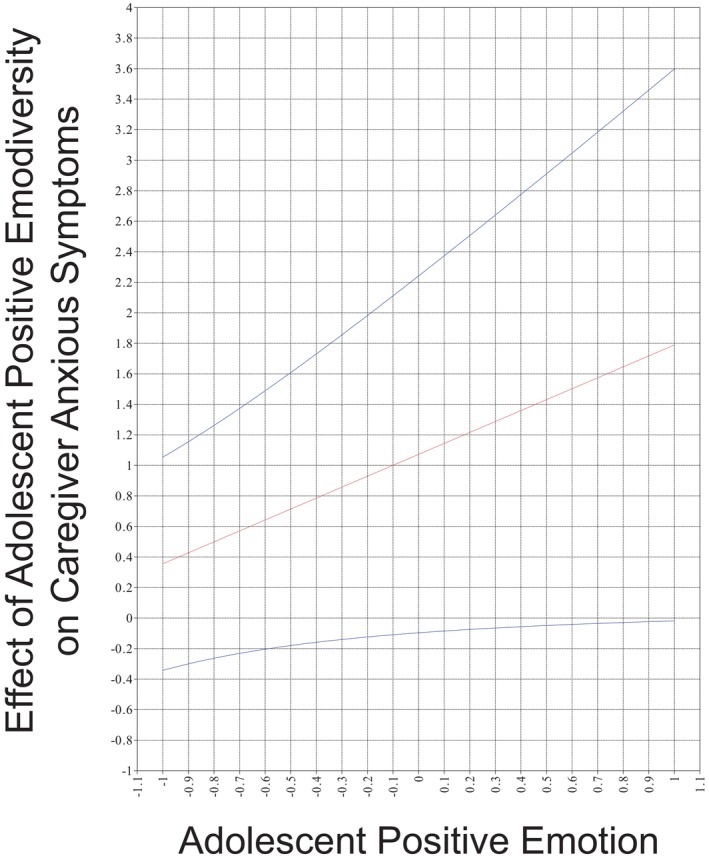
The interaction term of adolescent positive emotion and emodiversity predicting caregiver anxious symptoms. This plot represents the interaction between adolescent mean positive emotion and positive emodiversity on caregiver anxious symptoms. The red line represents the effect of adolescent positive emodiversity on caregiver anxious symptoms at different levels of adolescent mean positive emotion (x axis). Blue lines represent the 99% confidence intervals around this effect.

Greater adolescent mean positive emotion was associated with lower adolescent anxious symptoms. All other variables predicting adolescent anxious symptoms were not significant. In predicting caregiver anxious symptoms, lower levels of mean positive emotion were associated with greater caregiver anxious symptoms, whereas greater caregiver positive emodiversity was associated with greater anxious symptoms. There was a marginally significant (*p* = .055) interaction between adolescent mean positive emotion and positive emodiversity on caregiver anxious symptoms, which we cautiously interpreted given that the confidence interval did not contain 0. The positive effect of adolescent positive emodiversity on caregiver anxious symptoms was stronger at higher mean levels of adolescent positive emotion (see Figure 3). Regarding the multigroup analyses, greater levels of girls' mean positive emotions were associated with lower girls' anxious symptoms (see Table S7). For caregivers who participated with girls and boys, the positive actor effect of caregivers' positive emodiversity on their anxious symptom was greatest for caregivers with high mean levels of positive emotion (see Figures S1b and S1c). The positive effect of caregivers of boys' positive emodiversity on their own anxious symptoms was greatest at lower levels of caregivers' mean levels of positive emotion (see Figure S1d).

#### Model 3: Negative emotion variables and depressive symptoms

Greater adolescent negative emotion was associated with greater adolescent depressive symptoms, which was shown for both girls and boys in the multigroup analysis (see Table S8). All other variables predicting adolescent depressive symptoms were not significant. Greater caregiver mean negative emotion was associated with greater caregiver depressive symptoms. All other variables predicting caregiver depressive symptoms were not significant. Regarding the multigroup analysis, greater levels of both adolescent girls' and caregivers' own mean negative emotion were associated with greater caregiver depressive symptoms for caregivers who participated with girls. Lower levels of adolescent girls' negative emodiversity were also associated with greater caregiver depressive symptoms. For caregivers who participated with boys, greater boys' negative emodiversity was associated with greater caregiver depressive symptoms.

#### Model 4: Positive emotion variables and depressive symptoms

In predicting adolescent depressive symptoms, the significant main effect of adolescent positive emotion indicated that lower mean levels of positive emotion predicted greater depressive symptoms, as expected, which was also shown in multigroup analyses for girls and boys (see Table S9). All other predictors of adolescent depressive symptoms were not significant. In predicting caregiver depressive symptoms, there were significant main effects of caregivers' mean positive emotion and emodiversity. Lower mean positive emotion was associated with greater depressive symptoms, whereas greater positive emodiversity was associated with greater depressive symptoms. All other predictors were not significant. Regarding multigroup analysis, the positive effect of positive emodiversity on caregivers of girls' depressive symptoms was greatest at high levels of caregiver mean positive emotion (see Figure S1e). For caregivers who participated with boys, there was a significant main effect of caregivers' mean positive emotion, with lower levels being associated with greater caregiver depressive symptoms (see Table S9).

## DISCUSSION

We examined caregiver and adolescent negative and positive emotions in daily life (mean levels, emodiversity, and their interactions) in relation to anxious and depressive symptoms. For adolescents, greater negative emotions and lower positive emotions were associated with both anxious and depressive symptoms, as expected. Caregivers' emotions were not associated with adolescent internalizing symptoms. Results for caregiver internalizing symptoms were more complex. Greater caregiver negative emotions and lower positive emotions were associated with their anxious and depressive symptoms, as expected. Greater caregiver positive emodiversity was associated with greater anxious and depressive symptoms. Caregiver anxious symptoms were also marginally associated with adolescent positive emotions and emodiversity, where the positive association between adolescent positive emodiversity and caregiver anxious symptoms was greater at higher mean levels of adolescent positive emotion. Some potential differences by adolescent gender emerged, which we caution interpretation of given that these analyses were not preregistered and we have concerns that the sample size did not allow for robust examination of these potential differences. Taken together, these results add nuance to our understanding of how emotions experienced in daily life are linked to internalizing symptoms in the family context, which we elaborate on below.

### Associations among positive emotion, positive emodiversity, and internalizing symptoms

Our results highlight the importance of examining different aspects of positive emotions in daily life and their associations with different types of internalizing symptoms. Our findings that lower intensities of caregivers' and adolescents' own positive emotions were associated greater depressive symptoms is in line with the anhedonic difficulties that characterize depression (Bylsma et al., 2008). Mean levels of positive emotions were also associated with caregiver or adolescent anxious symptoms, in line with previous research indicating that positive emotions and their related processes may be disrupted in individuals experiencing anxiety (Carl et al., 2013). It seems that disruptions to positive emotions may be present in both middle‐aged adults and adolescents even at relatively low levels of depression.

Contrary to expectations, positive emodiversity was not associated with adolescents' own internalizing symptoms, but greater levels of caregivers' own positive emodiversity were associated with greater levels of their own anxious and depressive symptoms which was surprising. Previous research has shown that while positive emodiversity is associated with physical health (Ong et al., 2018), results are mixed regarding mental health. For example, some research has shown that positive emodiversity is associated with lower depressive symptoms (Quoidbach et al., 2014), whereas other research did not show any association between positive emodiversity and internalizing symptoms (Forster & Lougheed, 2024). More research is needed to determine when, and for whom, positive emodiversity is associated with mental health. In line with the perspective that a key feature of disrupted emotional functioning in depression is emotional context insensitivity (e.g., Bylsma et al., 2008), it is possible that positive emodiversity may only be associated with adolescent internalizing symptoms at higher levels of symptom severity than what was observed in the current study. For caregivers, low mean levels of positive emotion and greater positive emodiversity may reflect that some lability in positive emotion (i.e., low levels diverse in range) may be related to internalizing symptoms.

In terms of partner effects, adolescent positive emotion and emodiversity were associated with caregivers' internalizing symptoms. Specifically, the positive association between adolescent positive emodiversity and caregiver anxious symptoms were stronger at greater mean levels of adolescent positive emotion. In TIES, the associations between one person's emotion and the others' psychosocial adjustment are likely bidirectional across time scales, and multiple bursts of intensive longitudinal data collections are needed to fully test this (Lougheed, 2020). However, in the context of our cross‐sectional study, we put forth a possible explanation for this tentative finding. It could be that adolescents whose caregivers experience anxiety tend to up‐regulate both the intensity and diversity of their positive emotions in an attempt to ameliorate their caregivers' anxiety and to maintain the close and positive relationship dynamics with their caregivers that are a part of healthy relationships (Ramsey & Gentzler, 2015). An important future research direction is to disentangle the complex interrelations among caregivers' and adolescents' emotion dynamics and their associations with internalizing problems, including how caregivers' and adolescents' positive and negative emotions may be interrelated.

### Associations among negative emotion, negative emodiversity, and internalizing symptoms

In line with expectations, we observed that greater mean levels of adolescents' and caregivers' negative emotions were associated with greater levels of their own anxious and depressive symptoms. These actor effects are in line with decades of research indicating that anxious and depressive symptoms are both associated with elevated negative emotions (e.g., Campbell‐Sills et al., 2015; Joormann & Siemer, 2015).

None of the actor or partner effects of negative emodiversity on anxious and depressive symptoms were significant. These results contrast previous findings indicating that low negative emodiversity was associated with greater internalizing symptoms in the context of high mean negative emotion (Forster & Lougheed, 2024; Quoidbach et al., 2014). Although we prefer not to interpret null results, we speculate that our sample, which was relatively low in negative emotions and internalizing symptoms and high in socioeconomic status, may show a floor effect. We also draw attention to the consistently null findings that adolescent emodiversity was not associated with their internalizing problems.

### Limitations, future directions, and constraints on generalizability

Our study has several strengths, including the intensive longitudinal assessment in daily life, our dyadic statistical modeling approach, and our preregistered analyses. However, there are also some limitations that should be kept in mind while interpreting the results. First, there are some limitations regarding our study design. Our cross‐sectional design did not permit us to examine longitudinal associations between emotions and internalizing symptoms. Indeed, a multiple burst design is required to fully test how daily emotions and internalizing symptoms are linked across time scales within caregiver‐adolescent TIES (Lougheed, 2020). It would also be helpful for future research to include a measure of alexithymia (e.g., Parker et al., 2003) to determine to what extent emodiversity scores may or may not be related to individual differences that could impact responses to emotion questionnaires (Brown & Coyne, 2017; Quoidbach et al., 2018). In addition, we changed our study design twice during data collection in terms of total number of days and participant compensation. It is possible that the different study designs could have resulted in potential confounding factors, although we were not able to test this given the low cell sizes in our first two study designs (see Table S4 for descriptive statistics stratified by recruitment period).

Second, data from this sample may not generalize to other populations given the relatively high level of education and family income reported by caregivers, and the number of participating caregivers who identified as women. Future research on emodiversity in caregiver‐adolescent dyads is needed to explore these processes with other primary caregivers (e.g., fathers) and with samples more diverse in terms of educational background, ethnicity and cultural background, socioeconomic status, and gender identity. Our results may also not generalize across the full clinical range of internalizing symptoms. One crucial next step for research is to examine whether the associations between emodiversity and internalizing symptoms are nonlinear across the clinical spectrum (Bylsma et al., 2008). Given noted inconsistencies across research studies on the associations between emodiversity and mental health, this topic is a crucial next step for research in this area to disentangle when, and for whom, emodiversity is associated with mental health.

Third, it will be important for future research to examine whether the associations between emodiversity and internalizing problems vary by adolescent age. In our sample, we were not able to examine age‐related differences because we had a relatively restricted range of adolescent ages (13–17 years) and we were only able to collect age data in terms of numeric categories due to privacy concerns raised by the research ethics board on collecting personally‐identifying birthdates. Several forms of emotional variability (e.g., moment‐to‐moment flexibility, lability of specific emotions) play a role in adolescent internalizing symptoms (Maciejewski et al., 2014; Van der Giessen et al., 2014), and these associations may vary across adolescent development. Future research could examine longitudinal associations between emodiversity and internalizing symptoms in caregiver‐adolescent dyads with multiple burst designs (i.e., repeated assessments of intensive longitudinal data; Ram & Diehl, 2015) and/or examine age‐related differences in samples that include data on age as a continuous variable to examine these developmental questions directly.

In addition, although our sample size was likely too small to be able to result in a robust test of adolescent gender, our exploratory multigroup analyses point to some potentially complex patterns in how adolescent gender may be related to emotions, emodiversity, and internalizing symptoms. This topic area is key for future research, and we recommend that future work oversamples for diverse gender identities and includes a broader representation of caregiver gender identity so that this issue can be explored in full. Yet another important direction for future research is to examine these topics in clinical samples and/or samples that include individuals with greater levels of internalizing symptoms given that the associations between emotion‐related variables and internalizing symptoms may be non‐linear across levels of symptomatology (Bylsma, 2021).

### Conclusion

The current study demonstrates that the context of close family relationships such as between primary caregivers and adolescents is an important new direction for research on emodiversity. In line with TIES perspectives (Butler, 2011; Lougheed, 2020), we found interpersonal associations between adolescents' positive emotion levels and emodiversity may be related to caregivers' anxious symptoms, although these effects were not reciprocal. There are many fruitful future directions to understand how the richness of emotions experienced in daily life are related to internalizing symptoms in caregiver‐adolescent dyads.

## CONFLICT OF INTEREST STATEMENT

The authors have no conflicts of interest to disclose.

## Supporting information


Data S1.

## Data Availability

The data that support the findings of this study are openly available in Open Science Framework at https://osf.io/gkps8/, reference number DOI 10.17605/OSF.IO/GKPS8.
