# Supplementary material for: Associations between caregiver‐adolescent emodiversity and internalizing symptoms
Source: J Res Adolesc. 2024 Dec 1;35(1):e13041. doi: 10.1111/jora.13041 (PMC11758473; doi:10.1111/jora.13041)

**Associations between Caregiver-Adolescent Emodiversity and Internalizing Symptoms**

**Supplementary Material**

| **Table S1**  *Sample demographics* | | | |
| --- | --- | --- | --- |
|  | Adolescents (%) |  | Caregivers (%) |
| Gender identity |  |  |  |
| Boy/man | 44 |  | 6 |
| Girl/woman | 49 |  | 93 |
| Non-binary | 1 |  | 0 |
| Gender non-conforming | 1 |  | 0 |
| Questioning | 1 |  | 0 |
| Prefer not to disclose | 5 |  | 1 |
| Ethnicity |  |  |  |
| Endorsed multiple ethnicities | 33 |  | 15 |
| White | 39 |  | 53 |
| Chinese | 6 |  | 9 |
| South Asian & East Indian | 5 |  | 6 |
| Non-White Latin American | 4 |  | 6 |
| Black | 3 |  | 3 |
| Non-White West Asian | 2 |  | 2 |
| Japanese | 1 |  | 2 |
| Filipino | 1 |  | 2 |
| Korean | 1 |  | 1 |
| Non-White North African | 1 |  | 1 |
| Do not know | 1 |  | 0 |
| No response | 1 |  | 2 |
| Caregiver education |  |  |  |
| Graduate degree | — |  | 34 |
| Bachelor’s degree | — |  | 45 |
| Associate degree | — |  | 5 |
| Some college but no degree | — |  | 15 |
| High school degree or equivalent (GED) | — |  | 2 |
| No response | — |  | 1 |
| Household income (annual) |  |  |  |
| Above $150,000 | — |  | 33 |
| $100,000 to $150,000 | — |  | 31 |
| $75,000 to $100,000 | — |  | 17 |
| $50,000 to $75,000 | — |  | 8 |
| Less than $50,000 | — |  | 6 |
| No response | — |  | 6 |
| Caregiver relationship status |  |  |  |
| Married | — |  | 70 |
| Living with a domestic partner | — |  | 13 |
| Divorced | — |  | 7 |
| Separated | — |  | 6 |
| Single | — |  | 3 |
| Widowed | — |  | 3 |

| **Table S2**  *Correlations among study variables and demographic variables* | | | | | | |
| --- | --- | --- | --- | --- | --- | --- |
|  | Age | Gender | Ethnicity | Caregiver Education | Family Income | Caregiver Marital Status |
|  | Caregivers | | | | | |
| Mean Positive Emotion | 0.01 | — | -0.14 | -0.09 | 0.06 | 0.00 |
| Mean Negative Emotion | -0.26** | — | -0.02 | 0.12 | -0.25** | -0.10 |
| Positive Emotion Diversity | -0.10 | — | -0.13 | 0.03 | -0.04 | -0.01 |
| Negative Emotion Diversity | -0.15* | — | -0.01 | 0.03 | -0.19* | -0.12 |
| Depressive Symptoms | -0.06 | — | 0.05 | 0.14 | -0.09 | 0.00 |
| Anxious Symptoms | -0.18* | — | -0.01 | 0.06 | -0.27** | -0.12 |
|  | Adolescents | | | | | |
| Mean Positive Emotion | -0.01 | 0.34** | -0.07 | 0.04 | -0.10 | 0.04 |
| Mean Negative Emotion | 0.04 | -0.25** | 0.01 | -0.14 | 0.14 | -0.03 |
| Positive Emotion Diversity | 0.07 | 0.26** | -0.07 | 0.01 | 0.01 | 0.04 |
| Negative Emotion Diversity | 0.00 | -0.17* | 0.09 | -0.11 | 0.16* | -0.05 |
| Depressive Symptoms | -0.02 | -0.39** | -0.01 | -0.12 | 0.23* | 0.00 |
| Anxious Symptoms | -0.04 | -0.34** | -0.01 | -0.05 | 0.01 | 0.00 |
| *Note.* **p* < .05, ***p* < .001. Pearson correlations calculated between pairs of continuous variables and point-biserial correlations calculated between combinations of dichotomous and continuous variables. Caregiver Age treated as a continuous variable. Adolescent Age coded as 13-14 years = 0, 15-17 years = 1. Gender coded as Boy/Man = 1, Girl/Woman = 0, other genders = missing. Ethnicity coded as White = 1, Other = 0. Caregiver Education coded as 1 = Bachelor’s or graduate degree, Other = 0. Caregiver Marital Status coded as 1 = Married, 0 = Other. | | | | | | |

| **Table S4**  *Descriptive statistics by recruitment method* | | | | | |
| --- | --- | --- | --- | --- | --- |
|  | $26/21-Day Design  *M* (*SD*) |  | $26/14-Day Design  *M* (*SD*) |  | $35/14-Day Design  *M* (*SD*) |
| *n* dyads | 5 |  | 4 |  | 166 |
|  | Caregivers | | | | |
| Mean Positive Emotion | 1.96 (0.27) |  | 1.95 (0.67) |  | 2.16 (0.54) |
| Mean Negative Emotion | 0.64 (0.21) |  | 0.61 (0.31) |  | 0.67 (0.36) |
| Positive Emotion Diversity | 0.87 (0.06) |  | 0.89 (0.07) |  | 0.89 (0.07) |
| Negative Emotion Diversity | 0.56 (0.09) |  | 0.60 (0.11) |  | 0.59 (0.14) |
| Depressive Symptoms | 0.26 (0.08) |  | 1.10 (1.13) |  | 0.48 (0.41) |
| Anxious Symptoms | 0.31 (0.26) |  | 0.50 (0.31) |  | 0.40 (0.40) |
|  | Adolescents | | | | |
| Mean Positive Emotion | 1.92 (0.74) |  | 1.62 (0.92) |  | 2.06 (0.61) |
| Mean Negative Emotion | 0.96 (0.49) |  | 0.71 (0.31) |  | 0.90 (0.39) |
| Positive Emotion Diversity | 0.86 (0.07) |  | 0.72 (0.29) |  | 0.87 (0.08) |
| Negative Emotion Diversity | 0.74 (0.17) |  | 0.68 (0.17) |  | 0.72 (0.13) |
| Depressive Symptoms | 1.32 (0.27) |  | 1.10 (0.03) |  | 1.48 (0.30) |
| Anxious Symptoms | 0.55 (0.33) |  | 0.70 (0.56) |  | 0.89 (0.61) |

| **Table S3**  *Descriptive statistics by adolescent gender identity* | | | | | |  |  |
| --- | --- | --- | --- | --- | --- | --- | --- |
|  | Boy/Man  *M* (*SD*) |  | Girl/Woman  *M* (*SD*) |  | Questioning/ Gender non-conforming/ Nonbinary  *M* (*SD*) |  | Prefer not to respond  *M* (*SD*) |
| *n* adolescents | 77 |  | 85 |  | 4 |  | 8 |
| Mean Positive Emotion | 2.27 (0.58) |  | 1.86 (0.54) |  | 2.23 (0.57) |  | 1.44 (0.68) |
| Mean Negative Emotion | 0.80 (0.39) |  | 0.99 (0.37) |  | 0.94 (0.15) |  | 0.98 (0.52) |
| Positive Emotion Diversity | 0.89 (0.07) |  | 0.85 (0.09) |  | 0.85 (0.14) |  | 0.80 (0.21) |
| Negative Emotion Diversity | 0.70 (0.15) |  | 0.74 (0.11) |  | 0.81 (0.02) |  | 0.69 (0.14) |
| Depressive Symptoms | 1.34 (0.23) |  | 1.56 (0.30) |  | 1.79 (0.03) |  | 1.92 (0.49) |
| Anxious Symptoms | 0.65 (0.49) |  | 1.06 (0.63) |  | 0.89 (0.61) |  | 1.03 (0.78) |

| **Table S5** | | | | | | | | | | | | | | | | | | | | | | |
| --- | --- | --- | --- | --- | --- | --- | --- | --- | --- | --- | --- | --- | --- | --- | --- | --- | --- | --- | --- | --- | --- | --- |
| *Standardized Results for Actor-Partner Interdependence Models Predicting Internalizing Symptoms from Emotion and Emodiversity* | | | | | | | | | | | | | | | | | | | | | | |
| Model 1: Negative emotion and emodiversity predicting anxious symptoms | | | | | | | | | | | | | | | | | | | | | | |
|  | | | |  | |  | |  | | | |  | | | 95% CI | | | | | | | |
|  | | | |  | | Estimate | | *SE* | | | | *p* value | | | Low | | | High | | | | |
|  | | | |  | |  | |  | | | |  | | |  | | |  | | | | |
| Adolescent anxious symptoms | | | |  | |  | |  | | | |  | | |  | | |  | | | | |
| Adolescent mean negative emotion | | | | | 0.49 | | | 0.10 | | | | <.001 | | | 0.32 | | | 0.65 | | | | |
| Adolescent negative emodiversity | | | | | -0.02 | | | 0.11 | | | | .831 | | | -0.21 | | | 0.16 | | | | |
| Caregiver negative emotion | | | |  | | 0.24 | | 0.13 | | | | .061 | | | 0.03 | | | 0.45 | | | | |
| Caregiver negative emodiversity | | | |  | | -0.16 | | 0.11 | | | | .143 | | | -0.34 | | | 0.02 | | | | |
| Adolescent negative emotion x negative emodiversity | | | | | -0.06 | | | 0.07 | | | | .414 | | | -0.17 | | | 0.06 | | | | |
| Caregiver negative emotion x negative emodiversity | | | | | -0.07 | | | 0.09 | | | | .470 | | | -0.22 | | | 0.09 | | | | |
|  | | | |  | |  | |  | | | |  | | |  | | |  | | | | |
| Caregiver anxious symptoms | | | |  | |  | |  | | | |  | | |  | | |  | | | | |
| Adolescent mean negative emotion | | | | | 0.03 | | | 0.09 | | | | .700 | | | -0.11 | | | 0.18 | | | | |
| Adolescent negative emodiversity | | | | | 0.00 | | | 0.10 | | | | .965 | | | -0.17 | | | 0.16 | | | | |
| Caregiver negative emotion | | | |  | | 0.55 | | 0.12 | | | | <.001 | | | 0.35 | | | 0.75 | | | | |
| Caregiver negative emodiversity | | | |  | | 0.04 | | 0.11 | | | | .711 | | | -0.14 | | | 0.22 | | | | |
| Adolescent negative emotion x negative emodiversity | | | | | 0.07 | | | 0.07 | | | | .345 | | | -0.05 | | | 0.19 | | | | |
| Caregiver negative emotion x negative emodiversity | | | | | 0.01 | | | 0.09 | | | | .926 | | | -0.14 | | | 0.15 | | | | |
|  | | | |  | |  | |  | | | |  | | |  | | |  | | | | |
| Covariances | | | |  | |  | |  | | | |  | | |  | | |  | | | | |
| Adolescent negative emotion with: | | | | |  | | |  | | | |  | | |  | | |  | | | | |
| Adolescent negative emodiversity | | | | | 0.74 | | | 0.03 | | | | <.001 | | | 0.69 | | | 0.79 | | | | |
| Caregiver negative emotion | | | |  | | 0.13 | | 0.08 | | | | .102 | | | 0.00 | | | 0.25 | | | | |
| Caregiver negative emodiversity | | | |  | | 0.05 | | 0.09 | | | | .566 | | | -0.09 | | | 0.19 | | | | |
| Adolescent negative emotion x negative emodiversity | | | | | -0.01 | | | 0.13 | | | | .914 | | | -0.23 | | | 0.20 | | | | |
| Caregiver negative emotion x negative emodiversity | | | | | 0.02 | | | 0.08 | | | | .801 | | | -0.11 | | | 0.15 | | | | |
|  | | | |  | |  | |  | | | |  | | |  | | |  | | | | |
| Adolescent negative emodiversity with: | | | | |  | | |  | | | |  | | |  | | |  | | | | |
| Caregiver negative emotion | | | |  | | 0.14 | | 0.06 | | | | .025 | | | 0.04 | | | 0.24 | | | | |
| Caregiver negative emodiversity | | | |  | | 0.14 | | 0.07 | | | | .044 | | | 0.03 | | | 0.25 | | | | |
| Adolescent negative emotion x negative emodiversity | | | | | -0.37 | | | 0.12 | | | | .002 | | | -0.57 | | | -0.17 | | | | |
| Caregiver negative emotion x negative emodiversity | | | | | -0.01 | | | 0.06 | | | | .795 | | | -0.11 | | | 0.08 | | | | |
|  | | | |  | |  | |  | | | |  | | |  | | |  | | | | |
| Caregiver negative emotion with: | | | | |  | | |  | | | |  | | |  | | |  | | | | |
| Caregiver negative emodiversity | | | |  | | 0.72 | | 0.04 | | | | <.001 | | | 0.66 | | | 0.78 | | | | |
| Adolescent negative emotion x negative emodiversity | | | | | 0.00 | | | 0.06 | | | | .953 | | | -0.10 | | | 0.11 | | | | |
| Caregiver negative emotion x negative emodiversity | | | | | 0.51 | | | 0.12 | | | | <.001 | | | 0.32 | | | 0.71 | | | | |
|  | | | |  | |  | |  | | | |  | | |  | | |  | | | | |
| Caregiver negative emodiversity with: | | | | |  | | |  | | | |  | | |  | | |  | | | | |
| Adolescent negative emotion x negative emodiversity | | | | | -0.04 | | | 0.06 | | | | .490 | | | -0.14 | | | 0.06 | | | | |
| Caregiver negative emotion x negative emodiversity | | | | | 0.14 | | | 0.14 | | | | .347 | | | -0.10 | | | 0.37 | | | | |
|  | | | |  | |  | |  | | | |  | | |  | | |  | | | | |
| Adolescent negative emotion x negative emodiversity with: | | | | | | | |  | | | |  | | |  | | |  | | | | |
| Caregiver negative emotion x negative emodiversity | | | | | -0.01 | | | 0.06 | | | | .811 | | | -0.10 | | | 0.08 | | | | |
|  | | | |  | |  | |  | | | |  | | |  | | |  | | | | |
| Residual covariance between adolescent and caregiver anxious symptoms | | | | | 0.26 | | | 0.07 | | | | <.001 | | | 0.14 | | | 0.38 | | | | |
|  | | | |  | |  | |  | | | |  | | |  | | |  | | | | |
| Intercepts | | | |  | |  | |  | | | |  | | |  | | |  | | | | |
| Caregiver anxious symptoms | | | |  | | 0.99 | | 0.10 | | | | <.001 | | | 0.82 | | | 1.16 | | | | |
| Adolescent anxious symptoms | | | |  | | 1.51 | | 0.10 | | | | <.001 | | | 1.35 | | | 1.68 | | | | |
|  | | | |  | |  | |  | | | |  | | |  | | |  | | | | |
| Residual variances | | | |  | |  | |  | | | |  | | |  | | |  | | | | |
| Caregiver anxious symptoms | | | |  | | 0.65 | | 0.07 | | | | <.001 | | | 0.54 | | | 0.76 | | | | |
| Adolescent anxious symptoms | | | |  | | 0.74 | | 0.06 | | | | <.001 | | | 0.64 | | | 0.84 | | | | |
|  | | | |  | |  | |  | | | |  | | |  | | |  | | | | |
| Model 2: Positive emotions predicting anxious symptoms | | | | | | | | | | | | | | | | | | | | | |  |
|  | |  | | |  | | | | |  | | | |  | | | 95% *CI* | | | | |  |
|  | |  | | | Estimate | | | | | *SE* | | | | *p* value | | | Low | | High | | |  |
| Adolescent anxious symptoms | |  | | |  | | | | |  | | | |  | | |  | |  | | |  |
| Adolescent mean positive emotion | |  | | | -0.41 | | | | | 0.11 | | | | <.001 | | | -0.59 | | -0.22 | | |  |
| Adolescent positive emodiversity | |  | | | 0.15 | | | | | 0.16 | | | | .358 | | | -0.11 | | 0.40 | | |  |
| Caregiver positive emotion | |  | | | -0.07 | | | | | 0.11 | | | | .510 | | | -0.25 | | 0.11 | | |  |
| Caregiver positive emodiversity | |  | | | 0.00 | | | | | 0.12 | | | | .989 | | | -0.20 | | 0.20 | | |  |
| Adolescent positive emotion x positive emodiversity | | | | | -0.02 | | | | | 0.11 | | | | .858 | | | -0.19 | | 0.16 | | |  |
| Caregiver positive emotion x positive emodiversity | | | | | 0.13 | | | | | 0.10 | | | | .169 | | | -0.03 | | 0.29 | | |  |
|  | |  | | |  | | | | |  | | | |  | | |  | |  | | |  |
| Caregiver anxious symptoms | |  | | |  | | | | |  | | | |  | | |  | |  | | |  |
| Adolescent mean positive emotion | |  | | | -0.09 | | | | | 0.10 | | | | .355 | | | -0.26 | | 0.07 | | |  |
| Adolescent positive emodiversity | |  | | | 0.26 | | | | | 0.14 | | | | .064 | | | 0.03 | | 0.49 | | |  |
| Caregiver positive emotion | |  | | | -0.52 | | | | | 0.10 | | | | <.001 | | | -0.68 | | -0.35 | | |  |
| Caregiver positive emodiversity | |  | | | 0.32 | | | | | 0.11 | | | | .002 | | | 0.15 | | 0.50 | | |  |
| Adolescent positive emotion x positive emodiversity | | | | | 0.18 | | | | | 0.10 | | | | .055 | | | 0.03 | | 0.34 | | |  |
| Caregiver positive emotion x positive emodiversity | | | | | 0.09 | | | | | 0.09 | | | | .308 | | | -0.05 | | 0.23 | | |  |
|  | |  | | |  | | | | |  | | | |  | | |  | |  | | |  |
| Covariances | |  | | |  | | | | |  | | | |  | | |  | |  | | |  |
| Adolescent mean positive emotion with: | | | | |  | | | | |  | | | |  | | |  | |  | | |  |
| Adolescent positive emodiversity | |  | | | 0.69 | | | | | 0.04 | | | | <.001 | | | 0.63 | | 0.74 | | |  |
| Caregiver positive emotion | |  | | | 0.20 | | | | | 0.09 | | | | .022 | | | 0.06 | | 0.35 | | |  |
| Caregiver positive emodiversity | |  | | | 0.17 | | | | | 0.08 | | | | .038 | | | 0.04 | | 0.31 | | |  |
| Adolescent positive emotion x positive emodiversity | | | | | -0.25 | | | | | 0.12 | | | | .035 | | | -0.44 | | -0.06 | | |  |
| Caregiver positive emotion x positive emodiversity | | | | | -0.15 | | | | | 0.10 | | | | .135 | | | -0.31 | | 0.02 | | |  |
|  | |  | | |  | | | | |  | | | |  | | |  | |  | | |  |
| Adolescent positive emodiversity with: | | | | |  | | | | |  | | | |  | | |  | |  | | |  |
| Caregiver positive emotion | |  | | | 0.21 | | | | | 0.09 | | | | .022 | | | 0.06 | | 0.37 | | |  |
| Caregiver positive emodiversity | |  | | | 0.21 | | | | | 0.09 | | | | .019 | | | 0.06 | | 0.36 | | |  |
| Adolescent positive emotion x positive emodiversity | | | | | -0.67 | | | | | 0.11 | | | | <.001 | | | -0.85 | | -0.49 | | |  |
| Caregiver positive emotion x positive emodiversity | | | | | -0.19 | | | | | 0.14 | | | | .168 | | | -0.41 | | 0.04 | | |  |
|  | |  | | |  | | | | |  | | | |  | | |  | |  | | |  |
| Caregiver positive emotion with: | |  | | |  | | | | |  | | | |  | | |  | |  | | |  |
| Caregiver positive emodiversity | |  | | | 0.72 | | | | | 0.04 | | | | <.001 | | | 0.65 | | 0.79 | | |  |
| Adolescent positive emotion x positive emodiversity | | | | | -0.24 | | | | | 0.10 | | | | .015 | | | -0.41 | | -0.08 | | |  |
| Caregiver positive emotion x positive emodiversity | | | | | -0.33 | | | | | 0.13 | | | | .010 | | | -0.54 | | -0.12 | | |  |
|  | |  | | |  | | | | |  | | | |  | | |  | |  | | |  |
| Caregiver positive emodiversity with: | | | | |  | | | | |  | | | |  | | |  | |  | | |  |
| Adolescent positive emotion x positive emodiversity | | | | | -0.24 | | | | | 0.10 | | | | .017 | | | -0.41 | | -0.08 | | |  |
| Caregiver positive emotion x positive emodiversity | | | | | -0.55 | | | | | 0.10 | | | | <.001 | | | -0.70 | | -0.39 | | |  |
|  | |  | | |  | | | | |  | | | |  | | |  | |  | | |  |
| Adolescent positive emotion x positive emodiversity with: | | | | | | | | | |  | | | |  | | |  | |  | | |  |
| Caregiver positive emotion x positive emodiversity | | | | | 0.29 | | | | | 0.16 | | | | .082 | | | 0.02 | | 0.55 | | |  |
|  | |  | | |  | | | | |  | | | |  | | |  | |  | | |  |
| Residual covariance between adolescent and caregiver anxious symptoms | | | | | 0.27 | | | | | 0.07 | | | | <.001 | | | 0.16 | | 0.38 | | |  |
|  | |  | | |  | | | | |  | | | |  | | |  | |  | | |  |
| Intercepts | |  | | |  | | | | |  | | | |  | | |  | |  | | |  |
| Caregiver anxious symptoms | |  | | | 0.93 | | | | | 0.07 | | | | <.001 | | | 0.81 | | 1.05 | | |  |
| Adolescent anxious symptoms | |  | | | 1.39 | | | | | 0.09 | | | | <.001 | | | 1.25 | | 1.54 | | |  |
|  | |  | | |  | | | | |  | | | |  | | |  | |  | | |  |
| Residual variances: | |  | | |  | | | | |  | | | |  | | |  | |  | | |  |
| Caregiver anxious symptoms | |  | | | 0.83 | | | | | 0.05 | | | | <.001 | | | 0.75 | | 0.92 | | |  |
| Adolescent anxious symptoms | |  | | | 0.85 | | | | | 0.05 | | | | <.001 | | | 0.76 | | 0.94 | | |  |
|  | |  | | |  | | | | |  | | | |  | | |  | |  | | |  |
| Model 3: Negative emotions predicting depressive symptoms | | | | | | | | | | | | | | | | | | | | |  |  |
|  | | |  | | | |  | | | |  | |  | | | 95% *CI* | | | | |  |  |
|  | | |  | | | | Estimate | | | | *SE* | | *p* value | | | Low | | | High | |  |  |
|  | | |  | | | |  | |  | | | |  | | |  | | |  | |  |  |
| Adolescent depressive symptoms | | |  | | | |  | | | |  | |  | | |  | | |  | |  |  |
| Adolescent mean negative emotion | | |  | | | | 0.71 | | | | 0.12 | | <.001 | | | 0.51 | | | 0.92 | |  |  |
| Adolescent negative emodiversity | | |  | | | | -0.14 | | | | 0.17 | | .394 | | | -0.41 | | | 0.13 | |  |  |
| Caregiver negative emotion | | |  | | | | -0.09 | | | | 0.15 | | .564 | | | -0.33 | | | 0.16 | |  |  |
| Caregiver negative emodiversity | | |  | | | | 0.07 | | | | 0.11 | | .548 | | | -0.12 | | | 0.25 | |  |  |
| Adolescent negative emotion x negative emodiversity | | | | | | | -0.03 | | | | 0.11 | | .770 | | | -0.21 | | | 0.15 | |  |  |
| Caregiver negative emotion x negative emodiversity | | | | | | | 0.03 | | | | 0.08 | | .720 | | | -0.10 | | | 0.15 | |  |  |
|  | | |  | | | |  | | | |  | |  | | |  | | |  | |  |  |
| Caregiver depressive symptoms | | |  | | | |  | | | |  | |  | | |  | | |  | |  |  |
| Adolescent mean negative emotion | | |  | | | | 0.03 | | | | 0.11 | | .746 | | | -0.14 | | | 0.21 | |  |  |
| Adolescent negative emodiversity | | |  | | | | -0.01 | | | | 0.11 | | .951 | | | -0.20 | | | 0.18 | |  |  |
| Caregiver negative emotion | | |  | | | | 0.63 | | | | 0.13 | | <.001 | | | 0.42 | | | 0.83 | |  |  |
| Caregiver negative emodiversity | | |  | | | | 0.05 | | | | 0.10 | | .585 | | | -0.11 | | | 0.22 | |  |  |
| Adolescent negative emotion x negative emodiversity | | | | | | | 0.02 | | | | 0.07 | | .814 | | | -0.09 | | | 0.12 | |  |  |
| Caregiver negative emotion x negative emodiversity | | | | | | | -0.05 | | | | 0.06 | | .473 | | | -0.15 | | | 0.06 | |  |  |
|  | | |  | | | |  | | | |  | |  | | |  | | |  | |  |  |
| Covariances: | | |  | | | |  | | | |  | |  | | |  | | |  | |  |  |
| Adolescent mean negative emotion with: | | |  | | | |  | | | |  | |  | | |  | | |  | |  |  |
| Adolescent negative emodiversity | | |  | | | | 0.74 | | | | 0.03 | | <.001 | | | 0.69 | | | 0.79 | |  |  |
| Caregiver negative emotion | | |  | | | | 0.13 | | | | 0.08 | | .102 | | | 0.00 | | | 0.25 | |  |  |
| Caregiver negative emodiversity | | |  | | | | 0.05 | | | | 0.09 | | .566 | | | -0.09 | | | 0.19 | |  |  |
| Adolescent negative emotion x negative emodiversity | | | | | | | -0.02 | | | | 0.13 | | .911 | | | -0.23 | | | 0.20 | |  |  |
| Caregiver negative emotion x negative emodiversity | | | | | | | 0.02 | | | | 0.08 | | .801 | | | -0.11 | | | 0.15 | |  |  |
|  | | |  | | | |  | | | |  | |  | | |  | | |  | |  |  |
| Adolescent negative emodiversity with: | | |  | | | |  | | | |  | |  | | |  | | |  | |  |  |
| Caregiver negative emotion | | |  | | | | 0.14 | | | | 0.06 | | .025 | | | 0.04 | | | 0.24 | |  |  |
| Caregiver negative emodiversity | | |  | | | | 0.14 | | | | 0.07 | | .044 | | | 0.03 | | | 0.25 | |  |  |
| Adolescent negative emotion x negative emodiversity | | | | | | | -0.37 | | | | 0.12 | | .002 | | | -0.57 | | | -0.17 | |  |  |
| Caregiver negative emotion x negative emodiversity | | | | | | | -0.01 | | | | 0.06 | | .796 | | | -0.11 | | | 0.08 | |  |  |
|  | | |  | | | |  | | | |  | |  | | |  | | |  | |  |  |
| Caregiver negative emotion with: | | |  | | | |  | | | |  | |  | | |  | | |  | |  |  |
| Caregiver negative emodiversity | | |  | | | | 0.72 | | | | 0.04 | | <.001 | | | 0.66 | | | 0.78 | |  |  |
| Adolescent negative emotion x negative emodiversity | | | | | | | 0.00 | | | | 0.06 | | .956 | | | -0.10 | | | 0.11 | |  |  |
| Caregiver negative emotion x negative emodiversity | | | | | | | 0.51 | | | | 0.12 | | <.001 | | | 0.32 | | | 0.71 | |  |  |
|  | | |  | | | |  | | | |  | |  | | |  | | |  | |  |  |
| Caregiver negative emodiversity with: | | |  | | | |  | | | |  | |  | | |  | | |  | |  |  |
| Adolescent negative emotion x negative emodiversity | | | | | | | -0.04 | | | | 0.06 | | .481 | | | -0.14 | | | 0.06 | |  |  |
| Caregiver negative emotion x negative emodiversity | | | | | | | 0.14 | | | | 0.14 | | .347 | | | -0.10 | | | 0.37 | |  |  |
|  | | |  | | | |  | | | |  | |  | | |  | | |  | |  |  |
| Adolescent negative emotion x negative emodiversity with: | | | | | | | | | | |  | |  | | |  | | |  | |  |  |
| Caregiver negative emotion x negative emodiversity | | | | | | | -0.01 | | | | 0.06 | | .817 | | | -0.10 | | | 0.08 | |  |  |
|  | | |  | | | |  | | | |  | |  | | |  | | |  | |  |  |
| Residual covariance between adolescent and caregiver depressive symptoms | | | | | | | 0.05 | | | | 0.09 | | .627 | | | -0.11 | | | 0.20 | |  |  |
|  | | |  | | | |  | | | |  | |  | | |  | | |  | |  |  |
| Intercepts: | | |  | | | |  | | | |  | |  | | |  | | |  | |  |  |
| Caregiver depressive symptoms | | |  | | | | 1.19 | | | | 0.09 | | <.001 | | | 1.03 | | | 1.34 | |  |  |
| Adolescent depressive symptoms | | |  | | | | 4.98 | | | | 0.31 | | <.001 | | | 4.48 | | | 5.48 | |  |  |
|  | | |  | | | |  | | | |  | |  | | |  | | |  | |  |  |
| Residual variances: | | |  | | | |  | | | |  | |  | | |  | | |  | |  |  |
| Caregiver depressive symptoms | | |  | | | | 0.58 | | | | 0.07 | | <.001 | | | 0.47 | | | 0.69 | |  |  |
| Adolescent depressive symptoms | | |  | | | | 0.63 | | | | 0.07 | | <.001 | | | 0.51 | | | 0.74 | |  |  |
|  | | |  | | | |  | | | |  | |  | | |  | | |  | |  |  |
| Model 4: Positive emotions predicting depressive symptoms | | | | | | | | | | | | | | | | | | | |  |  |  |
|  |  | | | | | |  | | | |  | |  | | | 95% *CI* | | | |  |  |  |
|  |  | | | | | | Estimate | | | | *SE* | | *p* value | | | Low | | High | |  |  |  |
|  |  | | | | | |  | | | |  | |  | | |  | |  | |  |  |  |
| Adolescent depressive symptoms |  | | | | | |  | | | |  | |  | | |  | |  | |  |  |  |
| Adolescent mean positive emotion |  | | | | | | -0.75 | | | | 0.09 | | <.001 | | | -0.89 | | -0.60 | |  |  |  |
| Adolescent positive emodiversity |  | | | | | | 0.16 | | | | 0.11 | | .138 | | | -0.02 | | 0.34 | |  |  |  |
| Caregiver positive emotion |  | | | | | | 0.11 | | | | 0.09 | | .211 | | | -0.04 | | 0.26 | |  |  |  |
| Caregiver positive emodiversity |  | | | | | | 0.06 | | | | 0.09 | | .492 | | | -0.09 | | 0.21 | |  |  |  |
| Adolescent positive emotion x positive emodiversity | | | | | | | 0.12 | | | | 0.12 | | .296 | | | -0.07 | | 0.31 | |  |  |  |
| Caregiver positive emotion x positive emodiversity | | | | | | | 0.12 | | | | 0.09 | | .171 | | | -0.02 | | 0.26 | |  |  |  |
|  |  | | | | | |  | | | |  | |  | | |  | |  | |  |  |  |
| Caregiver depressive symptoms |  | | | | | |  | | | |  | |  | | |  | |  | |  |  |  |
| Adolescent mean positive emotion |  | | | | | | -0.05 | | | | 0.11 | | .636 | | | -0.23 | | 0.13 | |  |  |  |
| Adolescent positive emodiversity |  | | | | | | 0.10 | | | | 0.13 | | .471 | | | -0.12 | | 0.32 | |  |  |  |
| Caregiver positive emotion |  | | | | | | -0.55 | | | | 0.12 | | <.001 | | | -0.74 | | -0.36 | |  |  |  |
| Caregiver positive emodiversity |  | | | | | | 0.25 | | | | 0.11 | | .028 | | | 0.06 | | 0.44 | |  |  |  |
| Adolescent positive emotion x positive emodiversity | | | | | | | 0.26 | | | | 0.15 | | .094 | | | 0.00 | | 0.51 | |  |  |  |
| Caregiver positive emotion x positive emodiversity | | | | | | | 0.14 | | | | 0.10 | | .162 | | | -0.03 | | 0.31 | |  |  |  |
|  |  | | | | | |  | | | |  | |  | | |  | |  | |  |  |  |
| Adolescent mean positive emotion with: |  | | | | | |  | | | |  | |  | | |  | |  | |  |  |  |
| Adolescent positive emodiversity |  | | | | | | 0.69 | | | | 0.04 | | <.001 | | | 0.63 | | 0.74 | |  |  |  |
| Caregiver positive emotion |  | | | | | | 0.20 | | | | 0.09 | | .022 | | | 0.06 | | 0.35 | |  |  |  |
| Caregiver positive emodiversity |  | | | | | | 0.17 | | | | 0.08 | | .038 | | | 0.04 | | 0.31 | |  |  |  |
| Adolescent positive emotion x positive emodiversity | | | | | | | -0.25 | | | | 0.12 | | .035 | | | -0.44 | | -0.06 | |  |  |  |
| Caregiver positive emotion x positive emodiversity | | | | | | | -0.15 | | | | 0.10 | | .135 | | | -0.31 | | 0.02 | |  |  |  |
|  |  | | | | | |  | | | |  | |  | | |  | |  | |  |  |  |
| Adolescent positive emodiversity with: |  | | | | | |  | | | |  | |  | | |  | |  | |  |  |  |
| Caregiver positive emotion |  | | | | | | 0.21 | | | | 0.09 | | .022 | | | 0.06 | | 0.37 | |  |  |  |
| Caregiver positive emodiversity |  | | | | | | 0.21 | | | | 0.09 | | .019 | | | 0.06 | | 0.36 | |  |  |  |
| Adolescent positive emotion x positive emodiversity | | | | | | | -0.67 | | | | 0.11 | | <.001 | | | -0.85 | | -0.49 | |  |  |  |
| Caregiver positive emotion x positive emodiversity | | | | | | | -0.19 | | | | 0.14 | | .168 | | | -0.41 | | 0.04 | |  |  |  |
|  |  | | | | | |  | | | |  | |  | | |  | |  | |  |  |  |
| Caregiver positive emotion with: |  | | | | | |  | | | |  | |  | | |  | |  | |  |  |  |
| Caregiver positive emodiversity |  | | | | | | 0.72 | | | | 0.04 | | <.001 | | | 0.65 | | 0.79 | |  |  |  |
| Adolescent positive emotion x positive emodiversity | | | | | | | -0.24 | | | | 0.10 | | .015 | | | -0.41 | | -0.08 | |  |  |  |
| Caregiver positive emotion x positive emodiversity | | | | | | | -0.33 | | | | 0.13 | | .010 | | | -0.54 | | -0.12 | |  |  |  |
|  |  | | | | | |  | | | |  | |  | | |  | |  | |  |  |  |
| Caregiver positive emodiversity with: |  | | | | | |  | | | |  | |  | | |  | |  | |  |  |  |
| Adolescent positive emotion x positive emodiversity | | | | | | | -0.24 | | | | 0.10 | | .017 | | | -0.41 | | -0.08 | |  |  |  |
| Caregiver positive emotion x positive emodiversity | | | | | | | -0.55 | | | | 0.10 | | <.001 | | | -0.70 | | -0.39 | |  |  |  |
|  |  | | | | | |  | | | |  | |  | | |  | |  | |  |  |  |
| Adolescent positive emotion x positive emodiversity with: | | | | | | | | | | |  | |  | | |  | |  | |  |  |  |
| Caregiver positive emotion x positive emodiversity | | | | | | | 0.29 | | | | 0.16 | | .082 | | | 0.02 | | 0.55 | |  |  |  |
|  |  | | | | | |  | | | |  | |  | | |  | |  | |  |  |  |
| Residual covariance between adolescent and caregiver depressive symptoms | | | | | | | 0.15 | | | | 0.09 | | .118 | | | -0.01 | | 0.30 | |  |  |  |
|  |  | | | | | |  | | | |  | |  | | |  | |  | |  |  |  |
| Intercepts: |  | | | | | |  | | | |  | |  | | |  | |  | |  |  |  |
| Caregiver depressive symptoms |  | | | | | | 1.00 | | | | 0.10 | | <.001 | | | 0.84 | | 1.17 | |  |  |  |
| Adolescent depressive symptoms |  | | | | | | 4.77 | | | | 0.33 | | <.001 | | | 4.23 | | 5.32 | |  |  |  |
|  |  | | | | | |  | | | |  | |  | | |  | |  | |  |  |  |
| Residual variances: |  | | | | | |  | | | |  | |  | | |  | |  | |  |  |  |
| Caregiver depressive symptoms |  | | | | | | 0.71 | | | | 0.10 | | <.001 | | | 0.54 | | 0.87 | |  |  |  |
| Adolescent depressive symptoms |  | | | | | | 0.55 | | | | 0.08 | | <.001 | | | 0.42 | | 0.67 | |  |  |  |
| *Note.* *SE* = standard error, *CI* = confidence interval | | | | | | | | | | | | | | | | | | | |  |  |  |

***Actor and Partner Effects of Emotion on Internalizing Symptoms: Results from Multigroup Analyses Comparing Boys and Girls***

**Model 1:** **Negative Emotion Variables and Anxious Symptoms.** Results for Model 1 are shown in Table S6. There was a significant main effect of adolescent negative emotion for girls; specifically, greater mean negative emotion was associated with greater anxious symptoms. Adolescent girls’ anxious symptoms were also predicted by caregivers’ mean negative emotion, with greater levels being associated with greater anxious symptoms. All other variables predicting adolescent girls’ anxious symptoms were not significant, and all variables predicting adolescent boys’ anxious symptoms were not significant. In predicting caregiver anxious symptoms, there were significant main effects of caregiver negative emotion for caregivers who participated with girls and boys, with greater negative emotion being associated with greater caregiver anxious symptoms. All other variables predicting caregiver anxious symptoms were not significant for caregivers who participated with girls. In contrast, the significant main effect of adolescent negative emodiversity indicated that, for caregivers who participated with boys, greater boys’ negative emodiversity was associated with greater caregiver anxious symptoms. There was also a significant interaction between boys’ negative emodiversity and mean negative emotion in predicting caregivers’ anxious symptoms. Figure S1a shows this interaction effect, where the positive association of boys’ negative emodiversity on caregiver anxious symptoms was greater at higher levels of boys’ mean negative emotion. Thus, caregivers who participated with boys showed greater anxious symptoms when their boys showed high levels of mean negative emotion and high negative emodiversity. All other variables predicting caregiver anxious symptoms for caregivers who participated with boys were not significant.

**Model 2:** **Positive Emotion Variables and Anxious Symptoms.** Results for Model 2 are shown in Table S7. In predicting adolescent girls’ anxious symptoms, there was a significant main effect of adolescents’ mean positive emotion, where greater mean positive emotion was associated with lower anxious symptoms. All other variables predicting adolescent girls’ anxious symptoms were not significant. All variables predicting adolescent boys’ anxious symptoms were not significant. In predicting caregiver anxious symptoms for caregivers who participated with girls, there were significant main effects of caregivers’ mean positive emotion and positive emodiversity. Lower levels of mean positive emotion were associated with greater caregiver anxious symptoms, whereas greater caregiver positive emodiversity was associated with greater anxious symptoms. There was a significant interaction between caregivers’ mean positive emotion and positive emodiversity for caregivers who participated with girls. Figure S1b shows this interaction, where the positive association of caregivers’ positive emodiversity with their anxious symptoms was greatest for caregivers with high mean levels of positive emotion. For caregivers who participated with boys, significant partner effects indicated that greater mean levels of boys’ positive emotion were associated with lower caregiver anxious symptoms, whereas boys’ greater positive emodiversity was associated with greater caregiver anxious symptoms. There was a significant interaction between boys’ mean positive emotion and positive emodiversity. Figure S1c shows this interaction, where the positive effect of adolescent boys’ positive emodiversity on their caregivers’ anxious symptoms was greatest when boys also showed high mean levels of positive emotion. In terms of actor effects, caregivers who participated with boys showed a significant main effect of mean positive emotion, where lower levels of positive emotion were associated with greater anxious symptoms. There was a significant interaction between caregivers’ mean positive emotion and positive emodiversity. Figure S1d shows this interaction, where the positive effect of caregivers of boys’ positive emodiversity on their own anxious symptoms was greatest at lower levels of caregivers’ mean levels of positive emotion.

**Model 3: Negative Emotion Variables and Depressive Symptoms.** Results for Model 3 are shown in Table S8. For adolescent girls’ and boys’ depressive symptoms, there were significant main effects of adolescent mean negative emotion, indicating that greater mean negative emotion was associated with greater adolescent depressive symptoms. All other variables predicting adolescent girls’ and boys’ depressive symptoms were not significant. In predicting caregivers’ depressive symptoms, caregivers who participated with adolescent girls showed significant partner and actor effects of mean negative emotion, whereby greater levels of both adolescent girls’ and caregivers’ own mean negative emotion were associated with greater caregiver depressive symptoms. Lower levels of adolescent girls’ negative emodiversity were also associated with greater caregiver depressive symptoms, and all other variables predicting depressive symptoms of caregivers who participated with girls were not significant. For caregivers who participated with boys, greater boys’ negative emodiversity was associated with greater caregiver depressive symptoms. The significant main effect of caregivers’ own negative emotion indicated that greater mean levels of negative emotion were associated with greater caregiver depressive symptoms. All other variables predicting caregiver depressive symptoms were not significant.

**Model 4: Positive Emotion Variables and Depressive Symptoms.** Results for Model 4 are shown in Table S9. In predicting adolescent depressive symptoms, there was a significant main effect for both girls and boys of mean positive emotion, whereby lower mean positive emotion was associated with greater depressive symptoms. The significant main effect of caregivers’ positive emodiversity on boys’ depressive symptoms indicated that greater positive emodiversity was associated with lower depressive symptoms. All other variables predicting girls’ and boys’ depressive symptoms were not significant. In predicting depressive symptoms of caregivers who participated with girls, the significant main effect of caregivers’ positive emotion indicated that lower levels of positive emotion were associated with greater depressive symptoms. There was also a significant interaction between caregivers’ mean positive emotion and positive emodiversity. Figure S1e shows this interaction, where the positive effect of positive emodiversity on caregivers of girls’ depressive symptoms was greatest at high levels of caregiver mean positive emotion. All other variables predicting depressive symptoms of caregivers who participated with girls were not significant. For caregivers who participated with boys, there was a significant main effect of caregivers’ mean positive emotion, with lower levels being associated with greater caregiver depressive symptoms. All other variables predicting caregivers’ depressive symptoms for caregivers who participated with boys were not significant.

| **Table S6**  *Standardized Results for Multigroup Analyses of Actor-Partner Interdependence Model Predicting Anxious Symptoms from Negative Emotion and Emodiversity (Model 1)* | | | | | | | | | | | |
| --- | --- | --- | --- | --- | --- | --- | --- | --- | --- | --- | --- |
|  | Effect on Adolescent's Anxious Symptoms | | | | |  | Effect on Caregiver's Anxious Symptoms | | | | |
|  | β | *SE* | *p* | β 95% CI | |  | β | *SE* | *p* | β 95% CI | |
|  |  |  |  | Low | High |  |  |  |  | Low | High |
| Girls | | | | | | | | | | | |
| Adolescent Mean Negative Emotion | 0.48 | 0.12 | <.001 | 0.29 | 0.67 |  | 0.09 | 0.12 | .435 | -0.10 | 0.29 |
| Adolescent Negative Emodiversity | -0.02 | 0.11 | .853 | -0.20 | 0.16 |  | -0.11 | 0.12 | .380 | -0.31 | 0.09 |
| Adolescent Negative Emotion x Emodiversity | -0.09 | 0.09 | .288 | -0.23 | 0.05 |  | -0.03 | 0.11 | .786 | -0.20 | 0.14 |
| Caregiver Mean Negative Emotion | 0.34 | 0.15 | .024 | 0.09 | 0.59 |  | 0.50 | 0.16 | .002 | 0.24 | 0.75 |
| Caregiver Negative Emodiversity | -0.08 | 0.14 | .557 | -0.31 | 0.15 |  | 0.16 | 0.11 | .156 | -0.03 | 0.34 |
| Caregiver Negative Emotion x Emodiversity | -0.16 | 0.19 | .401 | -0.46 | 0.15 |  | -0.03 | 0.09 | .726 | -0.17 | 0.11 |
| Boys | | | | | | | | | | | |
|  | β | *SE* | *p* | β 95% CI | |  | β | *SE* | *p* | β 95% CI | |
|  |  |  |  | Low | High |  |  |  |  | Low | High |
| Adolescent Mean Negative Emotion | 0.25 | 0.21 | .224 | -0.09 | -0.27 |  | -0.04 | 0.15 | .797 | -0.28 | 0.21 |
| Adolescent Negative Emodiversity | 0.07 | 0.21 | .774 | -0.27 | 0.07 |  | 0.31 | 0.15 | .040 | 0.06 | 0.55 |
| Adolescent Negative Emotion x Emodiversity | -0.10 | 0.14 | .493 | -0.33 | 0.14 |  | 0.33 | 0.13 | .011 | 0.12 | 0.54 |
| Caregiver Mean Negative Emotion | 0.11 | 0.24 | .655 | -0.29 | 0.11 |  | 0.56 | 0.20 | .007 | 0.22 | 0.89 |
| Caregiver Negative Emodiversity | -0.16 | 0.22 | .477 | -0.53 | 0.21 |  | -0.07 | 0.19 | .697 | -0.39 | 0.24 |
| Caregiver Negative Emotion x Emodiversity | 0.14 | 0.13 | .257 | -0.07 | 0.35 |  | 0.10 | 0.13 | .451 | -0.12 | 0.31 |
| *Note. SE* = standard error, *CI* = confidence interval. | | | | | | | | | | | |

| **Table S7**  *Standardized Results for Multigroup Analyses of Actor-Partner Interdependence Model Predicting Anxious Symptoms from Positive Emotion and Emodiversity (Model 2)* | | | | | | | | | | | |
| --- | --- | --- | --- | --- | --- | --- | --- | --- | --- | --- | --- |
|  | Effect on Adolescent's Anxious Symptoms | | | | |  | Effect on Caregiver's Anxious Symptoms | | | | |
|  | β | *SE* | *p* | β 95% CI | |  | β | *SE* | *p* | β 95% CI | |
|  |  |  |  | Low | High |  |  |  |  | Low | High |
| Girls | | | | | | | | | | | |
| Adolescent Mean Positive Emotion | -0.31 | 0.15 | .031 | -0.55 | -0.07 |  | -0.04 | 0.17 | .829 | -0.31 | 0.24 |
| Adolescent Positive Emodiversity | 0.24 | 0.21 | .249 | -0.11 | 0.59 |  | 0.07 | 0.16 | .656 | -0.20 | 0.34 |
| Adolescent Positive Emotion x Emodiversity | 0.04 | 0.17 | .802 | -0.24 | 0.32 |  | -0.05 | 0.16 | .776 | -0.31 | 0.22 |
| Caregiver Mean Positive Emotion | -0.25 | 0.16 | .129 | -0.52 | 0.02 |  | -0.72 | 0.16 | <.001 | -0.99 | -0.46 |
| Caregiver Positive Emodiversity | -0.01 | 0.16 | .936 | -0.28 | 0.25 |  | 0.54 | 0.15 | <.001 | 0.30 | 0.78 |
| Caregiver Positive Emotion x Emodiversity | 0.03 | 0.16 | .834 | -0.23 | 0.30 |  | 0.27 | 0.14 | .045 | 0.05 | 0.49 |
| Boys | | | | | | | | | | | |
|  | β | *SE* | *p* | β 95% CI | |  | β | *SE* | *p* | β 95% CI | |
|  |  |  |  | Low | High |  |  |  |  | Low | High |
| Adolescent Mean Positive Emotion | -0.31 | 0.18 | .084 | -0.60 | -0.01 |  | -0.29 | 0.09 | .002 | -0.45 | -0.29 |
| Adolescent Positive Emodiversity | 0.12 | 0.21 | .570 | -0.23 | 0.47 |  | 0.51 | 0.12 | <.001 | 0.32 | 0.70 |
| Adolescent Positive Emotion x Emodiversity | -0.10 | 0.13 | .450 | -0.32 | 0.12 |  | 0.19 | 0.07 | .009 | 0.07 | 0.30 |
| Caregiver Mean Positive Emotion | 0.01 | 0.17 | .956 | -0.27 | 0.29 |  | -0.24 | 0.12 | .035 | -0.43 | -0.05 |
| Caregiver Positive Emodiversity | 0.17 | 0.18 | .358 | -0.13 | 0.47 |  | 0.21 | 0.13 | .091 | 0.01 | 0.42 |
| Caregiver Positive Emotion x Emodiversity | -0.04 | 0.15 | .804 | -0.27 | 0.20 |  | -0.24 | 0.06 | <.001 | -0.35 | -0.14 |
| *Note. SE* = standard error, *CI* = confidence interval. | | | | | | | | | | | |

| **Table S8**  *Standardized Results for Multigroup Analyses of Actor-Partner Interdependence Model Predicting Depressive Symptoms from Negative Emotion and Emodiversity (Model 3)* | | | | | | | | | | | |
| --- | --- | --- | --- | --- | --- | --- | --- | --- | --- | --- | --- |
|  | Effect on Adolescent's Depressive Symptoms | | | | |  | Effect on Caregiver's Depressive Symptoms | | | | |
|  | β | *SE* | *p* | β 95% CI | |  | β | *SE* | *p* | β 95% CI | |
|  |  |  |  | Low | High |  |  |  |  | Low | High |
| Girls | | | | | | | | | | | |
| Adolescent Mean Negative Emotion | 0.79 | 0.15 | <.001 | 0.54 | 1.03 |  | 0.37 | 0.11 | .001 | 0.18 | 0.56 |
| Adolescent Negative Emodiversity | -0.20 | 0.16 | 0.21 | -0.47 | 0.06 |  | -0.23 | 0.08 | .005 | -0.36 | -0.09 |
| Adolescent Negative Emotion x Emodiversity | -0.11 | 0.13 | .411 | -0.33 | 0.11 |  | -0.12 | 0.08 | 0.16 | -0.25 | 0.02 |
| Caregiver Negative Emotion | -0.11 | 0.21 | .606 | -0.46 | 0.24 |  | 0.69 | 0.13 | <.001 | 0.48 | 0.90 |
| Caregiver Negative Emodiversity | 0.30 | 0.16 | .056 | 0.04 | 0.57 |  | 0.13 | 0.10 | 0.22 | -0.04 | 0.30 |
| Caregiver Negative Emotion x Emodiversity | 0.00 | 0.11 | .982 | -0.18 | 0.18 |  | 0.03 | 0.08 | 0.70 | -0.10 | 0.15 |
| Boys | | | | | | | | | | | |
|  | β | *SE* | *p* | β 95% CI | |  | β | *SE* | *p* | β 95% CI | |
|  |  |  |  | Low | High |  |  |  |  | Low | High |
| Adolescent Mean Negative Emotion | 0.53 | 0.20 | .009 | 0.20 | 0.87 |  | -0.22 | 0.18 | .219 | -0.51 | 0.07 |
| Adolescent Negative Emodiversity | 0.06 | 0.24 | .792 | -0.34 | 0.47 |  | 0.50 | 0.16 | .002 | 0.23 | 0.77 |
| Adolescent Negative Emotion x Emodiversity | 0.08 | 0.19 | .664 | -0.24 | 0.40 |  | 0.19 | 0.11 | .079 | 0.01 | 0.37 |
| Caregiver Negative Emotion | -0.07 | 0.22 | .732 | -0.43 | 0.28 |  | 0.57 | 0.22 | .009 | 0.21 | 0.92 |
| Caregiver Negative Emodiversity | -0.09 | 0.17 | .604 | -0.38 | 0.20 |  | 0.04 | 0.17 | 0.24 | -0.24 | 0.32 |
| Caregiver Negative Emotion x Emodiversity | 0.13 | 0.13 | .296 | -0.08 | 0.34 |  | -0.20 | 0.11 | .074 | -0.38 | -0.02 |
| *Note. SE* = standard error, *CI* = confidence interval. | | | | | | | | | | | |

| **Table S9**  *Standardized Results for Multigroup Analyses of Actor-Partner Interdependence Model Predicting Depressive Symptoms from Positive Emotion and Emodiversity (Model 4)* | | | | | | | | | | | |
| --- | --- | --- | --- | --- | --- | --- | --- | --- | --- | --- | --- |
|  | Effect on Adolescent's Depressive Symptoms | | | | |  | Effect on Caregiver's Depressive Symptoms | | | | |
|  | β | *SE* | *p* | β 95% CI | |  | β | *SE* | *p* | β 95% CI | |
|  |  |  |  | Low | High |  |  |  |  | Low | High |
| Girls | | | | | | | | | | | |
| Adolescent Mean Positive Emotion | -0.58 | 0.16 | <.001 | -0.84 | -0.32 |  | -0.06 | 0.13 | .623 | -0.27 | 0.15 |
| Adolescent Positive Emodiversity | 0.21 | 0.23 | .356 | -0.16 | 0.58 |  | -0.05 | 0.19 | .796 | -0.35 | 0.26 |
| Adolescent Positive Emotion x Emodiversity | 0.21 | 0.24 | .386 | -0.19 | 0.60 |  | -0.09 | 0.13 | .519 | -0.31 | 0.13 |
| Caregiver Mean Positive Emotion | 0.20 | 0.16 | .218 | -0.07 | 0.47 |  | -0.63 | 0.20 | .002 | -0.97 | -0.30 |
| Caregiver Positive Emodiversity | -0.06 | 0.16 | .698 | -0.33 | 0.20 |  | 0.29 | 0.22 | .193 | -0.08 | 0.65 |
| Caregiver Positive Emotion x Emodiversity | 0.06 | 0.16 | .714 | -0.21 | 0.33 |  | 0.36 | 0.15 | .016 | 0.12 | 0.61 |
| Boys | | | | | | | | | | | |
|  | β | *SE* | *p* | β 95% CI | |  | β | *SE* | *p* | β 95% CI | |
|  |  |  |  | Low | High |  |  |  |  | Low | High |
| Adolescent Mean Positive Emotion | -0.77 | 0.09 | <.001 | -0.93 | -0.62 |  | -0.09 | 0.15 | .525 | -0.34 | 0.15 |
| Adolescent Positive Emodiversity | 0.08 | 0.10 | .427 | -0.08 | 0.24 |  | 0.17 | 0.15 | .255 | -0.08 | 0.42 |
| Adolescent Positive Emotion x Emodiversity | 0.08 | 0.05 | .122 | -0.01 | 0.16 |  | -0.04 | 0.10 | .718 | -0.21 | 0.13 |
| Caregiver Mean Positive Emotion | -0.11 | 0.10 | .300 | -0.28 | 0.06 |  | -0.40 | 0.17 | .016 | -0.68 | -0.13 |
| Caregiver Positive Emodiversity | 0.23 | 0.10 | .020 | 0.07 | 0.39 |  | 0.23 | 0.14 | .098 | 0.00 | 0.47 |
| Caregiver Positive Emotion x Emodiversity | 0.07 | 0.10 | .502 | -0.10 | 0.23 |  | -0.07 | 0.10 | .462 | -0.24 | 0.09 |
| *Note. SE* = standard error, *CI* = confidence interval. | | | | | | | | | | | |

**Figure S1.** Plots of the Significant Interaction Terms of Models 1, 2, and 4

*Note.* This plot represents the significant interactions from Models 1, 2, and 4. The Y axes represent the effects of emodiversity variables on internalizing symptoms. The X axes represent the mean levels of emotion variables. Red lines represent the effect of emodiversity on internalizing symptoms. Blue lines represent the 99% confidence intervals around these effects.


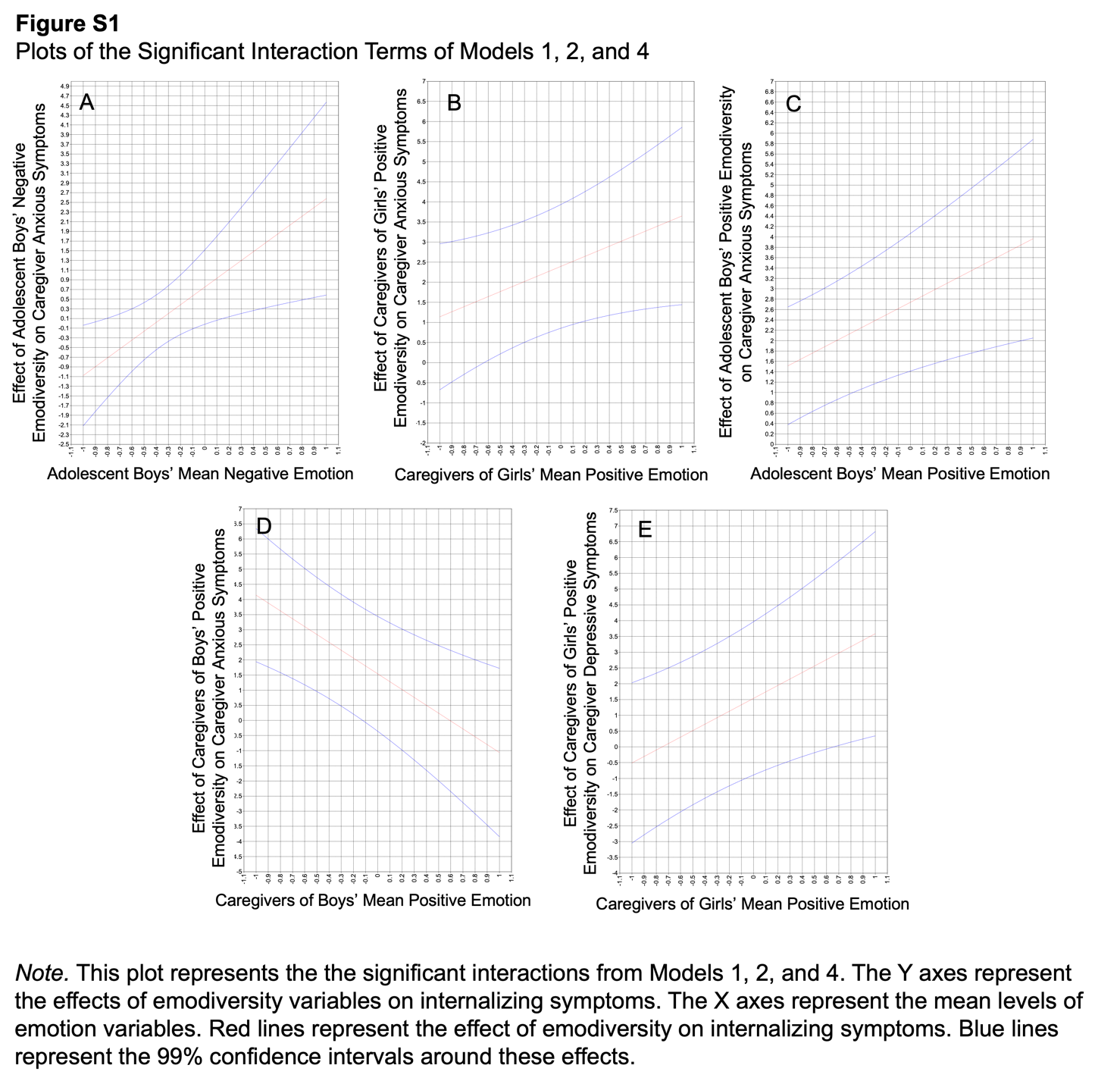

Supplement: Supplementary file 1 — Data S1. [file JORA-35-0-s001.docx]
